# Early B Cell and Plasma Cell Kinetics Upon Treatment Initiation Portend Flares in Systemic Lupus Erythematosus: A *Post-Hoc* Analysis of Three Phase III Clinical Trials of Belimumab

**DOI:** 10.3389/fimmu.2022.796508

**Published:** 2022-04-04

**Authors:** Ioannis Parodis, Alvaro Gomez, Jun Weng Chow, Alexander Borg, Julius Lindblom, Mariele Gatto

**Affiliations:** ^1^ Division of Rheumatology, Department of Medicine Solna, Karolinska Institutet and Karolinska University Hospital, Stockholm, Sweden; ^2^ Department of Rheumatology, Faculty of Medicine and Health, Örebro University, Örebro, Sweden; ^3^ Unit of Rheumatology, Department of Medicine, University of Padua, Padua, Italy

**Keywords:** systemic lupus erythematosus, biomarkers, flares, plasma cells, B cells, belimumab, biologics

## Abstract

**Objective:**

To investigate changes in B cell subsets in relation to disease flares upon initiation of standard therapy (ST) plus belimumab or placebo in patients with systemic lupus erythematosus (SLE).

**Patients and Methods:**

Using data from the BLISS-76, BLISS-SC and BLISS Northeast Asia trials, we investigated associations of relative to baseline rapid (through week 8) and early (through week 24) changes in peripheral B cell subsets, anti-dsDNA and complement levels with the occurrence of disease flares from week 24 through week 52 (Mann-Whitney *U* tests) or the entire study follow-up (Cox regression analysis), assessed using the SELENA-SLEDAI Flare Index.

**Results:**

Patients on ST alone who flared displayed less prominent early decreases in CD19^+^CD20^-^CD138^+^ long-lived plasma cells (-16.1% versus -35.1%; P=0.012). In all arms combined, patients who developed severe flares showed less prominent early decreases in CD19^+^CD20^-^CD138^+^ long-lived plasma cells (-23.5% versus -39.4%; P=0.028) and CD19^+^CD27^bright^CD38^bright^ SLE-associated plasma cells (-19.0% versus -27.8%; P=0.045). After adjustment for rapid changes, early increases in overall CD19^+^CD20^+^ B cells (HR: 1.81; 95% CI: 1.08–3.05; P=0.024) and early increases or no return after a rapid expansion in CD19^+^CD20^+^CD27^+^ memory B cells (HR: 1.58; 95% CI: 1.18–2.11; P=0.002) portended subsequent severe flares. Patients who developed flares of any severity showed no or less prominent rapid (0.0% versus -12.5%; P<0.001) or early (-1.9% versus -21.7%; P<0.001) decreases in anti-dsDNA levels, and patients who developed severe flares showed no or less prominent early decreases in anti-dsDNA levels (0.0% versus -13.3%; P=0.020). Changes in complement levels exhibited no ability to distinguish flaring from non-flaring patients.

**Conclusions:**

Increase or lack of decrease in certain circulating B cell subsets or anti-dsDNA levels upon treatment initiation for active SLE heralded subsequent severe disease flares. A rapid expansion of memory B cells may signify sustained response to therapy when followed by a subsequent drop, while no return or delayed increases in memory B cells may portend flaring. Peripheral B cell and serological marker kinetics may help identify patients in whom therapeutic modifications could protect against flare development, and may hence prove a useful complement to traditional surveillance and early treatment evaluation in SLE.

## 1 Introduction

Although the prognosis of patients with systemic lupus erythematosus (SLE) has improved during the last decades, occurrence of disease flares still endangers organ function and long-term outcomes (1–5), contributing to the burden of direct and indirect disease- and treatment-related morbidity and costs (6). Multiple definitions of flares have been proposed in SLE (7–9). Usually, flares are classified into mild/moderate or severe, based on the degree of therapeutic modification that is required and the impact on patient performance and eventually survival (10). To date, the risk of disease flares in patients with SLE is mainly determined based on short-term fluctuations of serological markers, which may show inconsistent results owing to different assays, time of sample collection, and the prominent heterogeneity in disease manifestations (11–13).

Belimumab blocks the soluble counterpart of B cell activating factor (BAFF; also known as B lymphocyte stimulator, BLyS) and has been used for the treatment of SLE for longer than a decade (14). Belimumab has shown ability to induce durable disease control and reduce the risk of flares in multiple clinical trials and real-life observational studies (15–21). However, early identification of patients at risk for subsequent flares upon commencement of belimumab treatment remains a challenge, leaving an area of uncertainty during the critical stages of early follow-up. This need was recently exemplified in a report of *de novo* lupus nephritis cases after initiation of belimumab therapy (22).

In this regard, biological changes occurring soon after treatment initiation might provide measurable tools that could be used to improve patient monitoring and stratification according to the risk for relapses. In this study, we aimed at investigating early changes in B cell and plasma cell subsets in relation to the development of disease flares during non-biological standard therapy (ST) plus belimumab or placebo within the frame of three phase III clinical trials of belimumab in SLE.

## 2 Patients and Methods

### 2.1 Study Population

We analysed longitudinal data from patients with active SLE who participated in three multicentre, randomised, double-blind, placebo-controlled trials comparing belimumab (administered intravenously or subcutaneously) with placebo, i.e., BLISS-76 (NCT00410384; N=797) (21), BLISS-SC (NCT01484496; N=822) (23), and BLISS Northeast Asia (NEA; NCT01345253; N=60) (24). The study population (N=1679) was selected based on availability of data on B cell subset counts and clinical data needed to determine flares. In the BLISS programmes, belimumab or placebo was administered on top of non-biological ST, including antimalarial agents, glucocorticoids, immunosuppressive agents, or combinations thereof.

In terms of design, the three trials were similar. Briefly, all patients were required to have a Safety of Estrogens in Lupus Erythematosus National Assessment - Systemic Lupus Erythematosus Disease Activity Index (SELENA-SLEDAI) (25) score ≥6 (BLISS-76) or ≥8 (BLISS-SC and BLISS-NEA) and had to be autoantibody positive (antinuclear antibody titres ≥1:80 and/or anti-double stranded (ds)DNA levels ≥30 IU/mL) at the screening. All patients had received stable dosages of ST for at least 30 days prior to baseline. For BLISS-76 and BLISS-NEA, belimumab or placebo were administered intravenously on days 0, 14, and 28, and every 4^th^ week thereafter through week 48 (BLISS-NEA) or week 72 (BLISS-76). The actual number of patients enrolled in BLISS-NEA was 702, and the selection of the 60 patients that were included in the present study was based on availability of B cell data from the initial trials. In BLISS-SC, belimumab 200 mg or placebo was administered subcutaneously weekly through week 52, on top of non-biological ST. Progressive restrictions were imposed during the trial periods on concurrent immunosuppressive and antimalarial medications, as well as glucocorticoid intake. The primary endpoint in all trials was the proportion of responders at week 52, with response being determined using the composite SLE Responder Index (SRI)-4 (26). The similar trial design and endpoints allowed pooling of the data to increase power during statistical analyses.

Occurrence of flares graded into mild/moderate or severe according to the SELENA-SLEDAI Flare Index (SFI) (10) was determined every fourth week.

### 2.2 Determination of B Cell Subsets and Serological Markers

Peripheral B cell and plasma cell subsets were determined by flow cytometry within the frame of the BLISS study programmes (21, 23, 24), and classified into total peripheral CD19^+^CD20^+^ B cells, CD19^+^CD20^+^CD69^+^ activated B cells, CD19^+^CD20^+^CD27^-^ naïve B cells, CD19^+^CD20^+^CD27^+^ memory B cells, CD19^+^CD20^-^CD27^bright^ plasmablasts, CD19^+^CD20^+^CD138^+^ short-lived plasma cells, CD19^+^CD20^-^CD138^+^ long-lived plasma cells, and CD19^+^CD38^bright^CD27^bright^ SLE-associated plasma cells (27–29). Levels of anti-dsDNA, C3 and C4 were determined within the frame of the BLISS programmes (21, 23, 24).

We analysed relative to baseline (i.e., treatment initiation) changes in B cell subsets and serum levels of anti-dsDNA, C3 and C4 that occurred through week 8, 24 and 52. Changes occurring through week 8 were deemed rapid and changes occurring through week 24 were deemed early. We next investigated associations between rapid or early changes in B cell or plasma cell subsets or changes in serological markers and flares occurring from week 24 through week 52 (Mann-Whitney *U* tests) or through the last observation (week 52 for BLISS-SC and BLISS-NEA, and week 76 for BLISS-76; Cox proportional hazards regression analysis).

### 2.3 Ethics

Data from the BLISS trials were made available by GlaxoSmithKline (Uxbridge, UK) through the Clinical Study Data Request (CSDR) consortium. The trial protocols were approved by regional ethics review boards for all participating centres and complied with the ethical principles of the Declaration of Helsinki. Written informed consent was obtained from all study participants prior to enrolment. The present study was approved by the Swedish Ethical Review Authority (2019-05498).

### 2.4 Statistical analysis

Descriptive statistics are reported as means and standard deviations or medians and interquartile ranges for continuous variables, while frequencies and percentages are reported for categorical variables. Values (relative to baseline percentage change) above the 97.5^th^ percentile were treated as extreme values and set to the same max value (equal to the 97.5^th^ percentile) for each cell variable.

Comparisons of distributions of the relative to baseline changes between groups (e.g., flaring versus non-flaring patients, or patients receiving belimumab versus placebo) were conducted using the non-parametric Mann-Whitney *U* test. For determination of time-dependent associations between rapid or early biological changes and flare occurrence, we used Cox proportional hazards regression models. All models were adjusted for age, sex, ethnicity, SLE disease duration, belimumab use (any dose), use of methotrexate, use of azathioprine, use of mycophenolate mofetil, use of immunosuppressants other than those mentioned before, and the BLISS study to account for batch variations in cell analyses. The potential interaction between cell alterations and belimumab use was accounted for. One set of models investigating associations between early B cell changes and flares occurring from week 24 through week 76 or the last available follow-up visit was also adjusted for the relative to baseline cell alterations from baseline through week 8 to account for alterations in opposing directions in the two follow-up phases.

P values below 0.05 were deemed significant. All analyses were performed using the R version 4.01 software (R Foundation for Statistical Computing, Vienna, Austria).

## 3 Results

### 3.1 Patient Characteristics

Demographics, clinical and serological data of the patients including comparisons between patients who developed and patients who did not develop flares (any grade or severe) are reported in **Table 1**. Baseline B cell and plasma cell data, including comparisons between patients who developed and patients who did not develop flares (any grade or severe) are reported in **Table 2**, where results are stratified by study to account for batch variations in cell analyses across studies.

**Table 1:** Characteristics of patients who developed versus patients who did not develop flares from week 24 through week 76 in the pooled BLISS study population.

	Any flare from week 24 through week 76				Severe flare from week 24 through week 76			
	All patients	Yes	No	P value	All patients	Yes	No	P value
	N=1533	N=959	N=574		N=1533	N=187	N=1346	
**Patient characteristics**								
**Age at baseline (years)**	39.3 ± 11.8	39.3 ± 11.4	39.3 ± 12.5	0.703	39.3 ± 11.8	38.8 ± 12.6	39.3 ± 11.7	0.461
**Female sex**	1439 (93.9%)	898 (93.6%)	541 (94.3%)	0.629	1439 (93.9%)	173 (92.5%)	1266 (94.1%)	0.410
**Ancestry**								
**Asian**	250 (16.3%)	133 (13.9%)	117 (20.4%)	**0.001**	250 (16.3%)	29 (15.5%)	221 (16.4%)	0.752
**Black/African American**	172 (11.2%)	125 (13.0%)	47 (8.2%)	**0.004**	172 (11.2%)	32 (17.1%)	140 (10.4%)	**0.006**
Indigenous American*	153 (10.0%)	108 (11.3%)	45 (7.8%)	**0.031**	153 (10.0%)	21 (11.2%)	132 (9.8%)	0.543
**White/Caucasian**	958 (62.5%)	593 (61.8%)	365 (63.6%)	0.492	958 (62.5%)	105 (56.1%)	853 (63.4%)	0.056
**Clinical data**								
**SLE duration at baseline (years)**	5.1 (1.7−10.6)	5.2 (1.7−10.6)	4.9 (1.5−10.8)	0.551	5.1 (1.7−10.6)	5.6 (2.3−11.1)	5.1 (1.6−10.5)	0.129
**Treatment at baseline**								
**Glucocorticoids**	1263 (82.4%)	747 (77.9%)	516 (89.9%)	**<0.001**	1263 (82.4%)	151 (80.7%)	1112 (82.6%)	0.530
**AMA^†^**	984 (64.2%)	626 (65.3%)	358 (62.4%)	0.251	984 (64.2%)	115 (61.5%)	869 (64.6%)	0.413
**Immunosuppressants^‡^**	787 (51.3%)	538 (56.1%)	249 (43.4%)	**<0.001**	787 (51.3%)	109 (58.3%)	678 (50.4%)	**0.042**
**Azathioprine**	301 (19.6%)	194 (20.2%)	107 (18.6%)	0.449	301 (19.6%)	43 (23.0%)	258 (19.2%)	0.217
**Methotrexate**	218 (14.2%)	159 (16.6%)	59 (10.3%)	**0.001**	218 (14.2%)	30 (16.0%)	188 (14.0%)	0.446
**Mycophenolate mofetil or sodium**	214 (14.0%)	156 (16.3%)	58 (10.1%)	**0.001**	214 (14.0%)	32 (17.1%)	182 (13.5%)	0.184
**Trial intervention**								
**Placebo**	505 (32.9%)	339 (35.3%)	166 (28.9%)	**0.010**	505 (32.9%)	82 (43.9%)	423 (31.4%)	**0.001**
**Belimumab**	1028 (67.1%)	620 (64.7%)	408 (71.1%)	**0.010**	1028 (67.1%)	105 (56.1%)	923 (68.6%)	**0.001**
**i.v. 1 mg/kg**	245 (16.0%)	186 (19.4%)	59 (10.3%)	**<0.001**	245 (16.0%)	31 (16.6%)	214 (15.9%)	0.812
**i.v. 10 mg/kg**	274 (17.9%)	193 (20.1%)	81 (14.1%)	**0.003**	274 (17.9%)	39 (20.9%)	235 (17.5%)	0.256
**s.c. 200 mg**	509 (33.2%)	241 (25.1%)	268 (46.7%)	**<0.001**	509 (33.2%)	35 (18.7%)	474 (35.2%)	**<0.001**
**Serological markers at baseline**								
**C3; mg/dL**	96.0 (75.0−118.5)	95.0 (73.0−119.0)	96.0 (77.0−117.0)	0.524	96.0 (75.0−118.5)	89.0 (64.0−110.0)	97.0 (76.0−119.0)	**<0.001**
**C4; mg/dL**	15.0 (9.0−22.0)	15.0 (9.0−22.0)	15.0 (9.0−21.0)	0.862	15.0 (9.0−22.0)	12.0 (7.0−19.0)	15.0 (9.0−22.0)	**0.001**
**anti-dsDNA; IU/mL (all patients)**	92.0 (29.0−275.0)	89.0 (29.0−285.0)	100.0 (29.0−268.3)	0.582	92.0 (29.0−275.0)	127.0 (29.0−429.0)	89.0 (29.0−254.3)	**0.002**
**anti-dsDNA; IU/mL (patients positive at baseline)**	162.0 (88.0−477.0); N=1045	167.0 (88.0−498.0); N=643	149.5 (86.0−426.0); N=402	0.443	162.0 (88.0−477.0); N=1045	245.0 (101.5−652.5); N=136	151.0 (86.0−450.5); N=909	**0.013**

Data are presented as number (percentage), mean ± standard deviation, or median (interquartile range), as appropriate. In case of missing values, the total number of patients with available data is indicated. Statistically significant P values are in bold.

*Alaska Native or American Indian from North, South or Central America.

^†^Hydroxychloroquine, chloroquine, mepacrine, mepacrine hydrochloride or quinine sulfate.

^‡^Azathioprine, cyclosporine, oral cyclophosphamide, leflunomide, methotrexate, mizoribine, mycophenolate mofetil, mycophenolate sodium or thalidomide.

AMA, antimalarial agents; C3, complement component 3; C4, complement component 4; i.v., intravenous; s.c., subcutaneous; SLE, systemic lupus erythematosus; SRI-4; SLE, Responder Index 4.

**Table 2:** B cell subset counts at baseline in patients who developed versus patient who did not develop flares from week 24 through week 76 in the BLISS-76, BLISS-SC and BLISS-NEA study population.

B cell subsets	All patients	Yes	No	P value
**BLISS-76**				
**Any flare from week 24 through week 76**				
	**N=720**	**N=553**	**N=167**	
**CD19^+^CD20^+^ (x10^3^/mL)**	91.5 (42.0−175.0); N=662	95.0 (42.3−175.0); N=504	81.0 (40.0−163.0); N=158	0.270
**CD19^+^CD20^+^CD27^+^ (x10^3^/mL)**	14.0 (6.0−27.0); N=662	14.5 (6.0−27.0); N=504	13.0 (7.0−25.0); N=158	0.464
**CD19^+^CD20^+^CD69^+^ (/mL)**	2096.5 (939.3−4357.5); N=650	2141.0 (867.5−4422.5); N=493	1958.0 (1010.0−4221.5); N=157	0.886
**CD19^+^CD20^+^CD27^-^ (x10^3^/mL)**	75.5 (32.8−141.3); N=662	79.0 (33.0−144.0); N=504	67.5 (30.5−127.0); N=158	0.209
**CD19^+^CD20^+^CD138^+^ (/mL)**	791.5 (329.3−1768.0); N=656	832.0 (357.0−1848.0); N=499	549.0 (263.5−1544.5); N=157	**0.014**
**CD19^+^CD20^-^CD138^+^ (/mL)**	474.0 (212.0−1059.0); N=655	485.0 (212.0−1083.0); N=499	449.0 (211.5−1040.0); N=156	0.931
**CD19^+^CD20^-^CD27^brt^ (/mL)**	312.0 (117.0−714.5); N=653	275.5 (107.0−668.3); N=496	456.0 (162.5−880.0); N=157	**0.004**
**CD19^+^CD27^brt^CD38^brt^ (/mL)**	320.0 (115.3−722.3); N=660	292.0 (109.8−675.5); N=502	438.0 (153.5−865.3); N=158	**0.008**
**Severe flare from week 24 through week 76**				
	**N=720**	**N=120**	**N=600**	
**CD19^+^CD20^+^ (x10^3^/mL)**	91.5 (42.0−175.0); N=662	91.0 (37.0−161.0); N=113	92.0 (43.0−175.5); N=549	0.463
**CD19^+^CD20^+^CD27^+^ (x10^3^/mL)**	14.0 (6.0−27.0); N=662	12.0 (5.0−26.5); N=113	15.0 (7.0−27.0); N=549	0.183
**CD19^+^CD20^+^CD69^+^ (/mL)**	2096.5 (939.3−4357.5); N=650	2385.0 (1063.3−5261.8); N=110	2046.5 (864.3−4296.3); N=540	0.196
**CD19^+^CD20^+^CD27^-^ (x10^3^/mL)**	75.5 (32.8−141.3); N=662	70.0 (30.0−136.5); N=113	76.0 (33.0−142.0); N=549	0.575
**CD19^+^CD20^+^CD138^+^ (/mL)**	791.5 (329.3−1768.0); N=656	756.0 (258.0−1961.0); N=113	795.0 (342.0−1696.0); N=543	0.942
**CD19^+^CD20^-^CD138^+^ (/mL)**	474.0 (212.0−1059.0); N=655	498.0 (209.0−1100.0); N=113	469.5 (211.8−1061.0); N=542	0.813
**CD19^+^CD20^-^CD27^brt^ (/mL)**	312.0 (117.0−714.5); N=653	274.5 (113.5−609.3); N=112	320.0 (119.0−743.5); N=541	0.480
**CD19^+^CD27^brt^CD38^brt^ (/mL)**	320.0 (115.3−722.3); N=660	285.0 (105.0−649.0); N=113	334.0 (120.0−732.0); N=547	0.274
**BLISS-SC**				
**Any flare from week 24 through week 76**				
	**N=757**	**N=377**	**N=380**	
**CD19^+^CD20^+^ (x10^3^/mL)**	107.0 (58.0−197.5); N=736	102.0 (53.0−189.0); N=363	108.0 (59.5−205.5); N=373	0.161
**CD19^+^CD20^+^CD27^+^ (x10^3^/mL)**	14.0 (7.0−29.0); N=736	12.0 (6.0−25.0); N=363	17.0 (7.0−32.0); N=373	**0.001**
**CD19^+^CD20^+^CD69^+^ (/mL)**	79.0 (32.0−198.8); N=736	74.0 (29.0−171.0); N=363	85.0 (35.0−230.0); N=373	**0.045**
**CD19^+^CD20^+^CD27^-^ (x10^3^/mL)**	89.0 (44.0−167.0); N=736	90.0 (43.0−158.0); N=363	89.0 (44.5−177.0); N=373	0.414
**CD19^+^CD20^+^CD138^+^ (/mL)**	53.0 (20.0−131.8); N=736	55.0 (22.0−130.0); N=363	52.0 (19.0−133.5); N=373	0.735
**CD19^+^CD20^-^CD138^+^ (/mL)**	198.0 (67.0−501.8); N=736	224.0 (69.0−566.0); N=363	176.0 (62.5−449.5); N=373	0.168
**CD19^+^CD20^-^CD27^brt^ (/mL)**	2000.0 (1000.0−4000.0); N=736	2000.0 (1000.0−4000.0); N=363	2000.0 (1000.0−4000.0); N=373	0.132
**CD19^+^CD27^brt^CD38^brt^ (/mL)**	1723.5 (728.3−3887.3); N=736	1594.0 (630.0−3733.0); N=363	1795.0 (763.0−4046.0); N=373	0.184
**Severe flare from week 24 through week 76**				
	**N=757**	**N=63**	**N=694**	
**CD19^+^CD20^+^ (x10^3^/mL)**	107.0 (58.0−197.5); N=736	70.0 (29.5−165.3); N=62	108.5 (60.8−200.0); N=674	**0.002**
**CD19^+^CD20^+^CD27^+^ (x10^3^/mL)**	14.0 (7.0−29.0); N=736	8.5 (5.0−21.3); N=62	15.0 (7.0−30.0); N=674	**0.001**
**CD19^+^CD20^+^CD69^+^ (/mL)**	79.0 (32.0−198.8); N=736	55.0 (26.0−111.0); N=62	82.0 (33.0−205.0); N=674	**0.007**
**CD19^+^CD20^+^CD27^-^ (x10^3^/mL)**	89.0 (44.0−167.0); N=736	61.0 (23.8−146.3); N=62	92.0 (46.0−170.3); N=674	**0.007**
**CD19^+^CD20^+^CD138^+^ (/mL)**	53.0 (20.0−131.8); N=736	44.0 (16.0−100.5); N=62	54.5 (20.0−135.0); N=674	0.155
**CD19^+^CD20^-^CD138^+^ (/mL)**	198.0 (67.0−501.8); N=736	248.0 (65.0−611.5); N=62	194.5 (67.0−496.5); N=674	0.460
**CD19^+^CD20^-^CD27^brt^ (/mL)**	2000.0 (1000.0−4000.0); N=736	1500.0 (750.0−3000.0); N=62	2000.0 (1000.0−4000.0); N=674	0.421
**CD19^+^CD27^brt^CD38^brt^ (/mL)**	1723.5 (728.3−3887.3); N=736	1698.5 (649.8−3620.0); N=62	1723.5 (728.8−3909.3); N=674	0.912
**BLISS NEA**				
**Any flare from week 24 through week 76**				
	**N=60**	**N=40**	**N=20**	
**CD19^+^CD20^+^ (x10^3^/mL)**	54.0 (22.0−102.0); N=51	54.0 (28.0−121.0); N=27	53.5 (17.3−90.5); N=24	0.503
**CD19^+^CD20^+^CD27^+^ (x10^3^/mL)**	7.4 (3.5−10.7); N=52	7.2 (3.2−11.7); N=28	7.4 (4.4−10.7); N=24	0.673
**CD19^+^CD20^+^CD69^+^ (/mL)**	106.6 (45.5−182.8); N=52	114.4 (46.8−182.8); N=28	106.6 (45.0−182.4); N=24	0.883
**CD19^+^CD20^+^CD27^-^ (x10^3^/mL)**	40.5 (18.7−94.5); N=52	43.1 (25.1−99.2); N=28	38.9 (15.0−77.8); N=24	0.533
**CD19^+^CD20^+^CD138^+^ (/mL)**	100.1 (58.3−247.3); N=52	84.9 (50.3−457.2); N=28	114.1 (64.1−201.3); N=24	0.783
**CD19^+^CD20^-^CD138^+^ (/mL)**	301.2 (175.6−685.7); N=52	390.5 (179.3−708.7); N=28	257.2 (128.1−596.8); N=24	0.322
**CD19^+^CD20^-^CD27^brt^ (/mL)**	970.6 (229.7−2204.8); N=52	1053.1 (290.2−2204.8); N=28	935.7 (213.4−2537.5); N=24	0.646
**CD19^+^CD27^brt^CD38^brt^ (/mL)**	954.4 (263.2−2218.4); N=52	998.5 (269.7−2218.4); N=28	919.8 (210.4−2274.6); N=24	0.633
**Severe flare from week 24 through week 76**				
	**N=56**	**N=4**	**N=52**	
**CD19^+^CD20^+^ (x10^3^/mL)**	54.0 (22.0−102.0); N=51	61.5 (14.3−158.3)	54.0 (28.0−95.0); N=47	0.879
**CD19^+^CD20^+^CD27^+^ (x10^3^/mL)**	7.4 (3.5−10.7); N=52	5.1 (3.6−59.6)	7.5 (3.5−10.7); N=48	0.882
**CD19^+^CD20^+^CD69^+^ (/mL)**	106.6 (45.5−182.8); N=52	100.6 (50.0−139.4)	106.6 (45.0−186.6); N=48	0.778
**CD19^+^CD20^+^CD27^-^ (x10^3^/mL)**	40.5 (18.7−94.5); N=52	57.2 (9.3−99.2)	40.5 (19.5−85.5); N=48	0.728
**CD19^+^CD20^+^CD138^+^ (/mL)**	100.1 (58.3−247.3); N=52	143.8 (40.8−442.3)	89.7 (58.3−247.3); N=48	0.753
**CD19^+^CD20^-^CD138^+^ (/mL)**	301.2 (175.6−685.7); N=52	173.4 (80.2−357.4)	309.6 (185.6−701.8); N=48	0.121
**CD19^+^CD20^-^CD27^brt^ (/mL)**	970.6 (229.7−2204.8); N=52	1059.9 (280.8−2322.3)	970.6 (229.7−2204.8); N=48	0.960
**CD19^+^CD27^brt^CD38^brt^ (/mL)**	954.4 (263.2−2218.4); N=52	1096.7 (296.8−2048.0)	954.4 (263.2−2308.9); N=48	1.000

Data are presented as medians (interquartile range) of absolute counts. In case of missing values, the total number of patients with available data is indicated. P values are derived from non-parametrical Mann-Whitney U tests. Statistically significant P values are in bold.

NEA, Northeast Asia; SC, subcutaneous.

### 3.2 Associations With Flares Occurring From Week 24 Through Week 52

#### 3.2.1 Flares of Any Severity (Mild/Moderate or Severe)

In the pooled datasets, 892/1533 patients (58.2%) developed at least one SFI flare of any degree of severity from week 24 through week 52. Among patients who flared, the first flare occurred after a mean time of 244.8 ± 61.0 days from baseline.

##### 3.2.1.1 B Cell Changes

**Figure 1:**
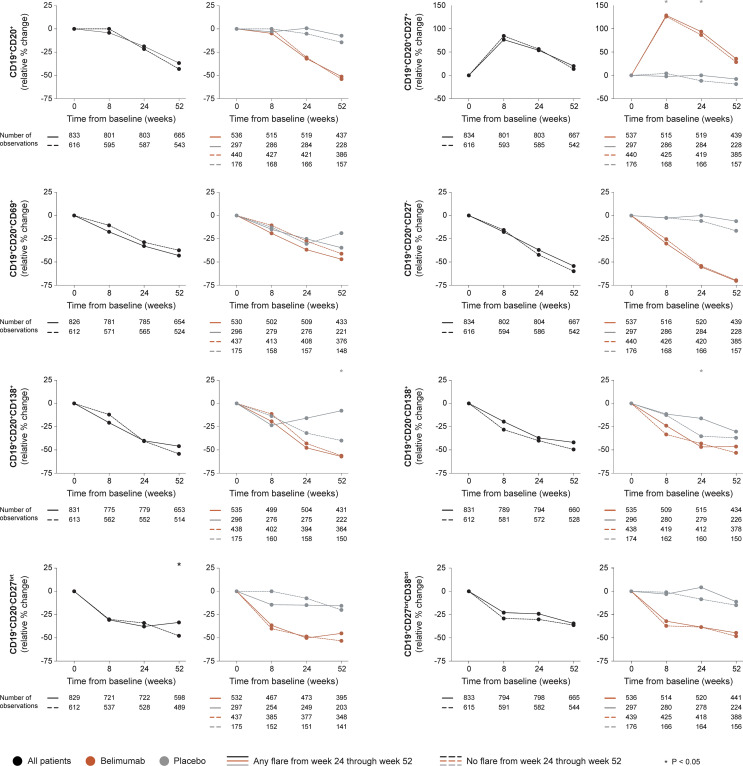
B cell alterations in relation to flares occurring from week 24 through week 52. The graphs delineate relative to baseline percentage changes in selected B cell and plasma cell subsets from baseline through different time points in patients who developed at least one SFI flare (mild/moderate or severe) from week 24 through week 52 (continuous lines) and patients who did not (dashed lines). Comparisons between patients who flared and patients who did not were conducted for the entire population with available data (black lines), and after stratification into patients who received standard therapy plus belimumab (terracotta lines) and patients who received standard therapy alone (grey lines). P values derived from non-parametric Mann-Whitney *U* tests. The number of patients with available data at each time point is indicated for each patient subgroup. SFI, Safety of Estrogens in Lupus Erythematosus National Assessment (SELENA) - Systemic Lupus Erythematosus Disease Activity Index (SLEDAI) Flare Index.

In the entire cohort (all treatment arms) and among patients who received add-on belimumab, no difference in rapid or early changes in any B cell subset was observed between patients who developed and patients who did not develop SFI flares of any severity from week 24 onwards (**Figure 1** and **Supplementary Tables S1**, **S2**). Among patients who received ST alone, patients who flared showed a slight decrease in CD19^+^CD20^+^CD27^+^ memory B cells through week 8 (-2.1%) while patients who did not flare showed an increase (+4.2%; P=0.037). Additionally, patients who flared exhibited less prominent decreases in CD19^+^CD20^-^CD138^+^ long-lived plasma cells from baseline through week 24 compared with patients who did not flare (-16.1% versus -35.1%; P=0.012). No difference was observed between flaring and non-flaring patients regarding rapid or early changes in CD19^+^CD20^+^ B cells (P=0.630 and P=0.082, respectively), CD19^+^CD20^+^CD69^+^ activated B cells (P=0.439 and P=0.681, respectively), CD19^+^CD20^-^CD27^bright^ plasmablasts (P=0.967 and P=0.772, respectively), or CD19^+^CD27^bright^CD38^bright^ SLE-associated plasma cells (P=0.681 and P=0.366, respectively).

##### 3.2.1.2 Serological Markers

**Figure 2:**
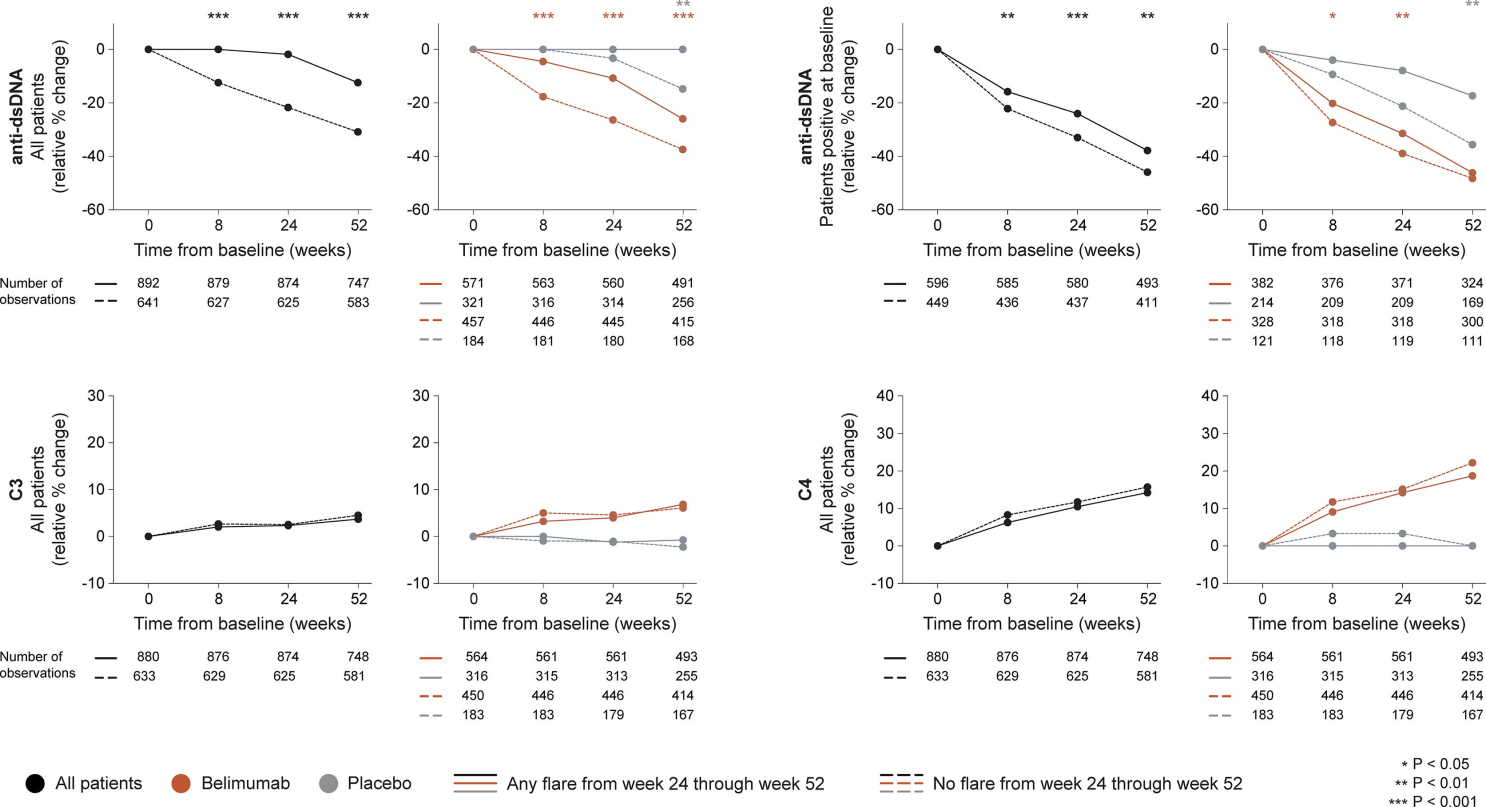
Changes in serological markers in relation to flares occurring from week 24 through week 52. The graphs delineate relative to baseline percentage changes in anti-dsDNA, C3 and C4 levels from baseline through different time points in patients who developed at least one SFI flare (mild/moderate or severe) from week 24 through week 52 (continuous lines) and patients who did not (dashed lines). Comparisons between patients who flared and patients who did not were conducted for the entire population with available data (black lines), and after stratification into patients who received standard therapy plus belimumab (terracotta lines) and patients who received standard therapy alone (grey lines). For anti-dsDNA levels, a separate analysis for patients with positive anti-dsDNA levels (≥30 IU/mL) at baseline is also demonstrated. P values derived from non-parametric Mann-Whitney *U* tests. The number of patients with available data at each time point is indicated for each patient subgroup. SFI, Safety of Estrogens in Lupus Erythematosus National Assessment (SELENA) - Systemic Lupus Erythematosus Disease Activity Index (SLEDAI) Flare Index; anti-dsDNA, anti-double stranded DNA antibodies; C3, complement component 3; C4, complement component 4.

In the entire cohort (all treatment arms), patients who developed flares of any severity from week 24 onwards showed no rapid change in anti-dsDNA levels (0.0%) while patients who did not flare showed rapid (-12.5%; P<0.001) and persistent decreases, which were consistently greater compared with those observed in flaring patients (baseline through week 24: -21.7% versus -1.9%; P<0.001), as well as in a subgroup analysis of patients with positive anti-dsDNA levels at baseline, both regarding rapid (through week 8; -22.2% versus -15.8%; P<0.001) and early changes (through week 24; -33.0% versus -24.0%; P<0.001). Changes in complement levels exhibited no ability to distinguish flaring from non-flaring patients. The results are illustrated in **Figure 2** and detailed in **Supplementary Tables S1**–**S3**.

Among patients who received add-on belimumab, patients who developed flares of any severity showed less prominent rapid (-4.6% versus -17.7%; P<0.001) and early (-10.8% versus -26.4%; P<0.001) relative to baseline decreases in anti-dsDNA levels compared with patients who did not flare, which was also the case in a subgroup analysis of patients with positive anti-dsDNA levels at baseline, both regarding rapid (-20.2% versus -27.4%; P=0.012) and early (-31.5% versus -39.0%; P=0.008) changes. No differences were observed regarding rapid or early changes in C3 or C4 levels (**Figure 2**).

Among patients who received ST alone, no differences were found between patients who flared and patients who did not flare from week 24 onwards regarding rapid or early changes in anti-dsDNA or complement levels.

#### 3.2.2 Severe Flares

In the pooled datasets, 163/1533 patients (10.6%) developed at least one severe flare from week 24 through week 52. Among patients who developed severe flares, the first severe flare occurred after a mean time of 253.6 ± 64.8 days from baseline.

##### 3.2.2.1 B Cell Changes

**Figure 3:**
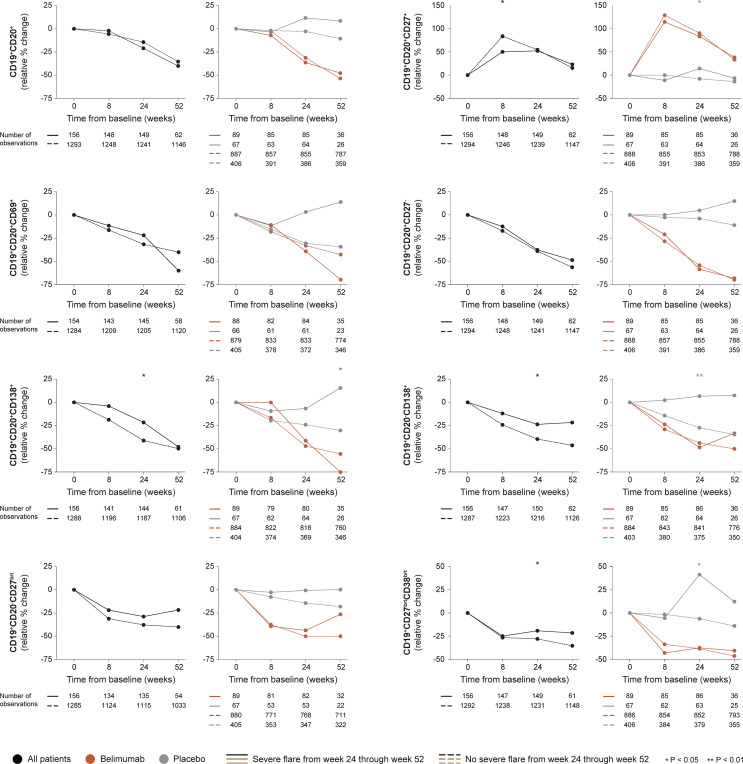
B cell alterations in relation to severe flares occurring from week 24 through week 52. The graphs delineate relative to baseline percentage changes in selected B cell and plasma cell subsets from baseline through different time points in patients who developed at least one severe SFI flare from week 24 through week 52 (continuous lines) and patients who did not (dashed lines). Comparisons between patients who flared and patients who did not were conducted for the entire population with available data (black lines), and after stratification into patients who received standard therapy plus belimumab (terracotta lines) and patients who received standard therapy alone (grey lines). P values derived from non-parametric Mann-Whitney *U* tests. The number of patients with available data at each time point is indicated for each patient subgroup. SFI, Safety of Estrogens in Lupus Erythematosus National Assessment (SELENA) - Systemic Lupus Erythematosus Disease Activity Index (SLEDAI) Flare Index.

In the entire cohort (all treatment arms), patients who developed at least one severe flare from week 24 onwards showed less prominent rapid increases through week 8 in CD19^+^CD20^+^CD27^+^ memory B cells compared with patients who did not develop severe flares (+50.0% versus +83.5%; P=0.037), as shown in **Figure 3**. Furthermore, patients who developed severe flares displayed less prominent relative to baseline decreases through week 24 in CD19^+^CD20^-^CD138^+^ long-lived plasma cells (-23.5% versus -39.4%; P=0.028), CD19^+^CD20^+^CD138^+^ short-lived plasma cells (21.5% versus -41.1%; P=0.024) and CD19^+^CD27^bright^CD38^bright^ SLE-associated plasma cells (-19.0% versus -27.8%; P=0.045) compared with patients who did not develop severe flares. No differences were observed between patients who developed severe flares compared with patients who did not regarding rapid or early changes in CD19^+^CD20^+^ B cells (P=0.967 and P=0.323, respectively), CD19^+^CD20^+^CD69^+^ activated B cells (P=0.378 and P=0.431, respectively) or CD19^+^CD20^+^CD27^-^ naïve B cells (P=0.273 and P=0.313, respectively), or rapid changes in CD19^+^CD20^+^CD138^+^ short-lived plasma cells (P=0.599). The results are delineated in **Figure 3** and detailed in **Supplementary Tables S1**–**S3**.

Among patients who received add-on belimumab, no differences in rapid or early changes across any B cell subset were observed between patients who developed severe flares and patients who did not.

Among patients who received non-biological ST alone, patients who developed severe flares showed an increase while patients who did not develop severe flares showed a decrease from baseline through week 24 in CD19^+^CD20^+^CD27^+^ memory B cells (+14.3% versus -7.7%; P=0.023), CD19^+^CD20^-^CD138^+^ long-lived plasma cells (+6.7% versus -27.2%; P=0.002) and CD19^+^CD27^bright^CD38^bright^ SLE-associated plasma cells (+41.2% versus -6.1%; P=0.038), resulting in a significant difference in all cases. No difference was observed between patients who developed severe flares and patients who did not regarding rapid or early changes in the overall CD19^+^CD20^+^ B cell pool (P=0.972 and P=0.062, respectively), CD19^+^CD20^+^CD69^+^ activated B cells (P=0.653 and P=0.159, respectively), CD19^+^CD20^+^CD27^-^ naïve B cells (P=0.761 and P=0.101, respectively), CD19^+^CD20^-^CD27^bright^ plasmablasts (P=0.272 and P=0.184, respectively), or CD19^+^CD20^+^CD138^+^ short-lived plasma cells (P=0.755 and P=0.106, respectively; **Figure 3**).

##### 3.2.2.2 Serological Markers

**Figure 4:**
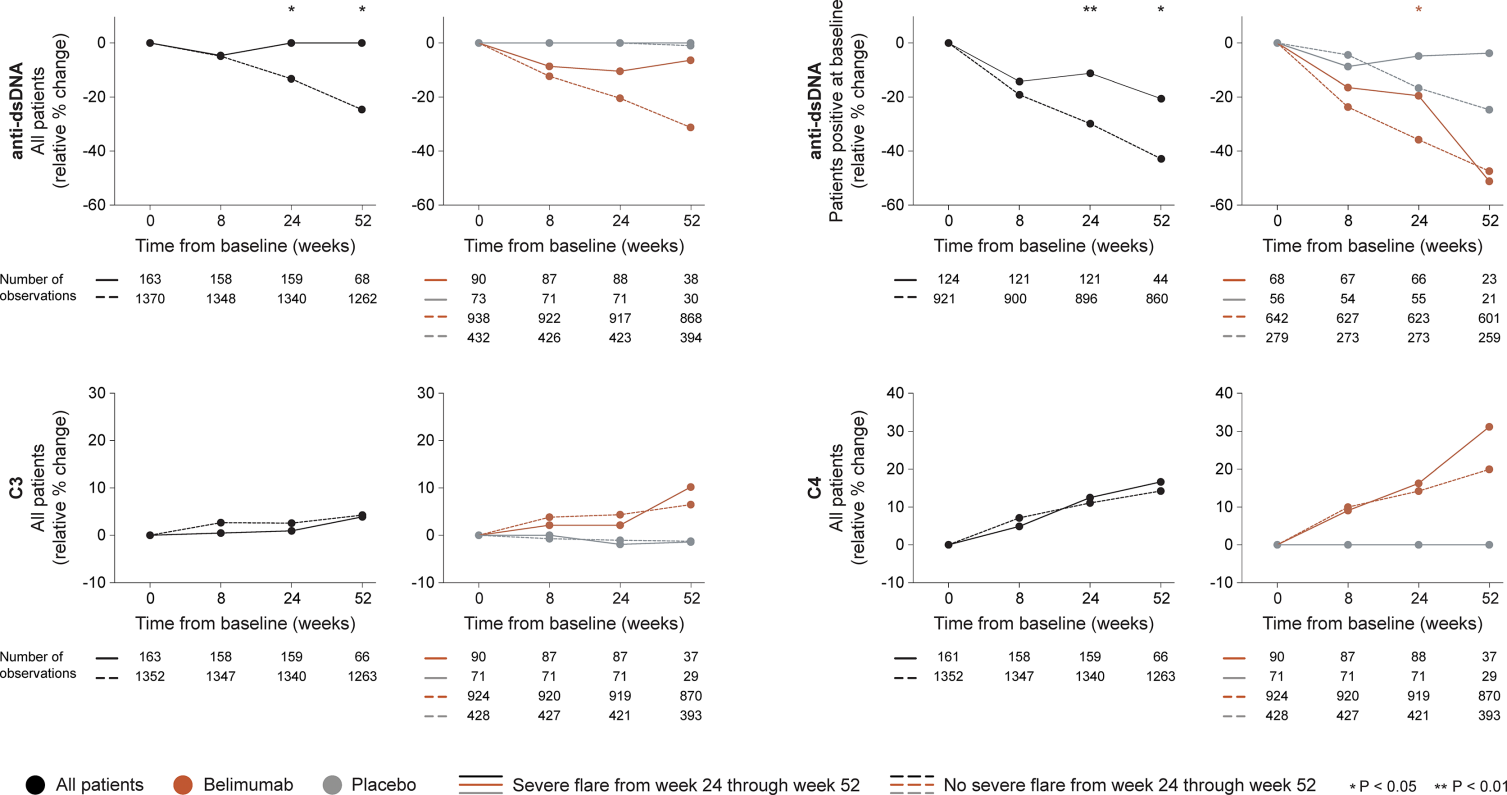
Changes in serological markers in relation to severe flares occurring from week 24 through week 52. The graphs delineate relative to baseline percentage changes in anti-dsDNA, C3 and C4 levels from baseline through different time points in patients who developed at least one severe SFI flare from week 24 through week 52 (continuous lines) and patients who did not (dashed lines). Comparisons between patients who flared and patients who did not were conducted for the entire population with available data (black lines), and after stratification into patients who received standard therapy plus belimumab (terracotta lines) and patients who received standard therapy alone (grey lines). For anti-dsDNA levels, a separate analysis for patients with positive anti-dsDNA levels (≥30 IU/mL) at baseline is also demonstrated. P values derived from non-parametric Mann-Whitney *U* tests. The number of patients with available data at each time point is indicated for each patient subgroup. SFI, Safety of Estrogens in Lupus Erythematosus National Assessment (SELENA) - Systemic Lupus Erythematosus Disease Activity Index (SLEDAI) Flare Index; anti-dsDNA, anti-double stranded DNA antibodies; C3, complement component 3; C4, complement component 4.

In the entire cohort (all treatment arms), no differences between patients who developed severe flares and patients who did not were documented regarding rapid changes in anti-dsDNA or complement levels. Patients who developed at least one severe flare from week 24 onwards showed no early change (0.0%) while patients who did not develop severe flares exhibited early decreases in anti-dsDNA levels (-13.3%; P=0.020). In a subgroup analysis of patients with positive anti-dsDNA levels at baseline, the relative to baseline decrease in anti-dsDNA levels through week 24 was less prominent in patients who developed severe flares from week 24 onwards compared with patients who did not (-11.2% versus -29.8%; P=0.003), as shown in **Figure 4**. No differences between patients who developed severe flares and patients who did not were seen regarding early changes in C3 or C4 levels (**Figure 4**).

A similar pattern was seen among patients who received add-on belimumab. Patients who developed at least one severe flare from week 24 onwards showed a trend towards less prominent decreases in anti-dsDNA levels through week 24 compared with patients who did not develop severe flares (-10.5 versus -20.5%), which however did not reach statistical significance (P=0.071). Nevertheless, in the subgroup analysis of patients with positive anti-dsDNA levels at baseline, the decreases in anti-dsDNA levels through week 24 were less prominent in patients who developed severe flares from week 24 onwards compared with patients who did not (-19.6% versus -35.9%; P=0.022). No differences between patients who developed severe flares and patients who did not were seen regarding rapid or early changes in C3 or C4 levels (**Figure 4**).

Among patients who received non-biological ST alone, no differences between patients who developed severe flares and patients who did not were seen regarding rapid or early relative to baseline changes in anti-dsDNA, C3 or C4 levels (**Figure 4**).

### 3.3 Associations With Disease Flares in Time-Dependent Cox Regression Models

#### 3.3.1 Flares of Any Severity (Mild/Moderate or Severe)

In the pooled datasets, 959/1533 patients (62.6%) developed at least one SFI flare of any degree of severity from week 24 through the end of the study period (week 52 in BLISS-SC and BLISS-NEA; week 76 in BLISS-76). Among patients who flared, the first flare occurred after a mean time of 254.4 ± 76.7 days from baseline.

**Figure 5:**
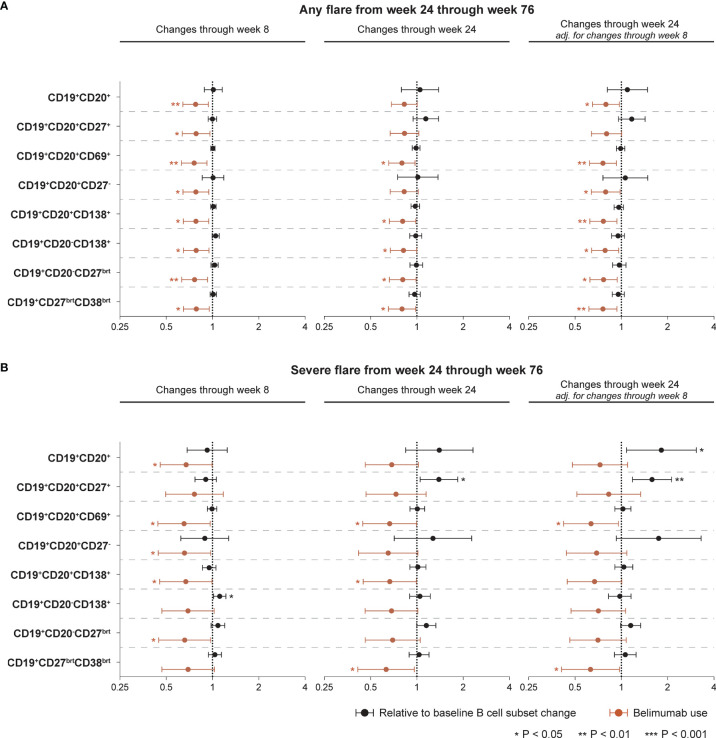
Associations between B cell alterations and flare development. The forest plots illustrate results from proportional hazards (Cox) regression analysis, investigating associations between rapid or early relative to baseline percentage changes in selected B cell and plasma cell subsets and development of the first SFI flare of any severity (mild/moderate or severe; **(A)** or the first severe SFI flare **(B)** occurring from week 24 through week 76 or the last available follow-up visit. All models included belimumab use (any dose) as a covariate, and the result for the respective model is plotted in terracotta colour. The potential interaction between cell alterations and belimumab use were accounted for. Additionally, all models were adjusted for age, sex, ethnicity, SLE disease duration, use of methotrexate, use of azathioprine, use of mycophenolate mofetil, use of immunosuppressants other than those mentioned before, and the BLISS study to account for batch variations in cell analyses. One set of models investigating associations between early B cell changes and flare development was also adjusted for the relative to baseline cell alterations from baseline through week 8 to account for alterations in opposing directions in the rapid and early follow-up phase. Circles denote hazard ratios and whiskers denote 95% confidence intervals. Statistically significant associations are indicated with asterisks. SFI, Safety of Estrogens in Lupus Erythematosus National Assessment (SELENA) - Systemic Lupus Erythematosus Disease Activity Index (SLEDAI) Flare Index.

Proportional hazards (Cox) regression models showed no ability of alterations in B cell or plasma cell subsets to portend flares of any severity occurring from week 24 onwards in the entire study population, being the case for both rapid changes through week 8 and early changes through week 24, the latter also in models adjusted for the rapid phase B cell changes (**Figure 5A**). By contrast, use of belimumab was shown to be overall protective against disease flares. The results are detailed in **Supplementary Table S4**, including the interaction term between belimumab use and relative to baseline B cell changes. Thus, the hazard ratio (HR) of flare development in belimumab-treated patients is derived by multiplication of the HR for the interaction term with the HR for B cell changes in the respective model. Regarding flares of any severity, the interaction term did not reach statistical significance in any model.

#### 3.3.2 Severe Flares

In the pooled datasets, 187/1533 patients (12.2%) developed at least one severe flare from week 24 through the end of the study period. Among patients who developed severe flares, the first severe flare occurred after a mean time of 274.3 ± 88.4 days from baseline.

Rapid increases in CD19^+^CD20^-^CD138^+^ long-lived plasma cells from baseline through week 8 were associated with a higher likelihood and/or shorter time to the first severe flare from week 24 onwards (HR: 1.11; 95% CI: 1.01–1.22; P=0.024), while changes in the other B cell or plasma cell subsets during the rapid phase exhibited no significant association with development of severe flares. Add-on belimumab was shown to exert an overall protective effect, which however did not reach significance in the models of CD19^+^CD20^+^CD27^+^ memory B cells, CD19^+^CD20^-^CD138^+^ long-lived plasma cells and CD19^+^CD27^bright^CD38^bright^ SLE-associated plasma cells (**Figure 5B**; **Supplementary Table S4**).

Notably, early increases in CD19^+^CD20^+^CD27^+^ memory B cells from baseline through week 24 were associated with a higher likelihood and/or shorter time to the first severe flare from week 24 onwards, both before (HR: 1.39; 95% CI: 1.05–1.84; P=0.022) and after (HR: 1.58; 95% CI: 1.18–2.11; P=0.002) adjustment for changes in CD19^+^CD20^+^CD27^+^ memory B cells during the rapid phase (from baseline through week 8), while add-on belimumab showed no protective effect in these models (**Figure 5B**). The interaction term between belimumab use and relative to baseline changes in CD19^+^CD20^+^CD27^+^ memory B cells through week 24 was statistically significant (HR:0.72; 95% CI: 0.52−0.99; P=0.044) in the unadjusted model for the changes through week 8. Thus, relative to baseline changes in CD19^+^CD20^+^CD27^+^ memory B cells through week 24 were associated with a 39% increased hazard of subsequent severe flare development when the patient was on placebo, while for belimumab-treated patients this hazard was minimal (1.39 x 0.72 = 1.0008), in line with the unadjusted analysis presented in **Figure 3**. Changes in the other B cell or plasma cell subsets from baseline through week 24 exhibited no significant association with development of severe flares before adjustment for the rapid phase. Following adjustment for the rapid phase, early increases in the total CD19^+^CD20^+^ B cell pool were associated with a higher likelihood and/or shorter time to the first severe flare (HR: 1.81; 95% CI: 1.08–3.05; P=0.024; **Figure 5B**). The results are detailed in **Supplementary Table S4**.

## 4 Discussion

In this paper, we analysed data from three phase III clinical trials of SLE. We demonstrated that increasing trends in long-lived plasma cells during an initial rapid phase and in memory B cells during a later intermediate phase upon commencement of therapy with belimumab or placebo on top of non-biological ST were associated with subsequent severe flares. Our study introduces dynamics in peripheral B cell and plasma cell subsets as a potential complemental tool in the surveillance of lupus patients. It is worth noting that among patients treated with add-on belimumab, patients who developed flares exhibited more modest decreases in anti-dsDNA levels compared with patients who did not flare, providing important implications about the potential usefulness of anti-dsDNA dynamics in early evaluation of belimumab therapy. To the best of our knowledge, this is the first documentation of the relationship between rapid and early changes in circulating B lymphocyte subsets and subsequent disease flares in a large SLE population, with potential implications regarding surveillance strategies and early treatment evaluation in patients with SLE.

Prevention of flares is included among SLE treatment goals, since they may heavily influence the patients’ prognosis, e.g., by contributing to organ damage accrual and morbidity (30, 31). Unfortunately, despite advanced therapeutics during the last decades (14), implementation of efficient preventive strategies remains an unmet need, and flares are not rare even upon treatment initiation (32, 33), the latter *per se* thus not constituting a guarantee for disease quiescence. Moreover, early determination of the risk for disease flares in patients commencing treatment for active SLE is still not feasible in clinical practice. Considering the important role of B cells in SLE (30), exploration of the relationship between their kinetics upon treatment initiation and disease flaring is intriguing.

Following the advent of the anti-BAFF biological agent belimumab, several studies have highlighted that this drug reduces the burden of flares in patients with SLE (2, 16–18). Considering its mode of action, belimumab is expected to hamper the survival of B cells, especially immature B cells, which has been corroborated in previous research (28, 34–36). Thus, declining B cell subsets, especially B cell subsets of early developmental stages, could be expected to signify better responses to belimumab therapy, in a similar manner as successful B cell depletion has been shown to be coupled with good responses to treatment with rituximab (37, 38).

Our hypothesis was that prominent biological changes towards abatement of B cell activity upon therapy initiation would be associated with a protection against flares, and since biological changes have been shown to precede the measurable clinical improvement induced by belimumab (34), one could expect that alterations in B cell subsets in patients who are protected from flares occur early after treatment initiation. The concept of monitoring early biological changes to portend therapeutic outcome should not be regarded as contradicting that of baseline predictors, but rather complemental towards optimised surveillance, early and efficient decision-making, and better outcomes. For instance, serological status at baseline has been shown to be informative regarding the outcome of belimumab therapy (39, 40), as have early decreases in levels of interleukin (IL)-6 (41).

Flares may occur at any time during patient follow-up and have been reported both as an early and a delayed event upon treatment initiation (3, 5, 17). In the present study, we assessed changes in peripheral B cell and plasma cell subsets preceding disease flares which occurred from week 24 from baseline and throughout a follow-up of up to 76 weeks. We showed that more prominent rapid and early decreases in long-lived plasma cells were inversely associated with subsequent flares, particularly severe flares. Stratification of patients by treatment arm (ST plus belimumab and ST alone) revealed that the inverse association between early decreases in long-lived plasma cells and subsequent flaring was significant in patients who received non-biological ST alone in unadjusted analysis but not in patients who received add-on belimumab. Thus, early and profound decreases in long-lived plasma cells may signify greater expected drug efficacy and a protective effect against flares when broad immunosuppression is commenced, whereas belimumab may rather be expected to induce decreases irrespective of the treatment outcome. While this observation should be interpreted with caution since it was not replicated in the Cox regression analysis for the early treatment phase, it has some interest in light of inconsistent results in previous research regarding the impact of belimumab therapy on plasma cell subsets (28, 34–36). In this respect, the large study population and the investigation of several distinct plasma cell subsets carried out in the present study may have facilitated the detection of subsets within the plasma cell pool, the kinetics of which may have particular prognostic value.

By contrast, a rapid increase in memory B cells was found to be inversely associated with subsequent occurrence of severe flares. Interestingly, however, a later relative to baseline increase in memory B cells through week 24 was also shown to portend severe flares in time-dependent Cox regression analysis. This seemingly conflicting finding becomes interesting in light of knowledge that belimumab therapy induces an early expansion of memory B cells, with a subsequent return towards baseline values (35, 36), which however has not been put in relation to a longer-term treatment outcome. The findings herein imply that while this initial expansion may be associated with belimumab efficacy and a lower likelihood to develop severe flares, the lack of return or a continued increase in memory B cells may be associated with abatement of the drug efficacy and flare development. Following stratification by treatment arms, the rapid expansion of memory B cells was evidently driven by belimumab, although the numbers were not sufficient to demonstrate significant differences between flaring and non-flaring patients within treatment groups. Notably, it was also evident that among patients who received non-biological ST alone, those who developed severe flares from week 24 onwards displayed an increase in memory B cells through week 24 following an initial drop, whereas belimumab-treated patients displayed a rapid increase in circulating memory B cells followed by a subsequent return regardless of flare occurrence.

Importantly, relative increases in the overall B cell pool through week 24 were also found to herald subsequent severe flares in Cox regression analysis, however only after adjustment for B cell changes during the rapid treatment phase, which complicates the interpretation of this finding. The link between changes in the circulating B cell pool and clinical response has been investigated in response to anti-CD20 treatment in SLE and lupus nephritis, with overall depletion of B cells being associated with better responses (38, 42), whereas a quick repopulation of memory B cells and plasmablasts heralded lupus flares (37). Our findings yield further merit to the concept of B cell monitoring as a relevant tool for patient follow-up upon therapy, especially B cell modulatory therapy, and provide novel implications of a connection between changes in distinct B cell and plasma cell subsets in the periphery following anti-BAFF treatment and occurrence of lupus flares.

We also investigated changes in anti-dsDNA and complement levels. In this analysis, anti-dsDNA antibody levels decreased more prominently through week 24 in belimumab-treated patients who did not develop subsequent severe flares compared with belimumab-treated patients who developed severe flares, whereas in placebo-treated patients this difference reached significance only at week 52. This corroborates the known usefulness of anti-dsDNA antibodies in surveillance of patients with SLE (43, 44), here also in the context of treatment evaluation (45), especially early evaluation of treatment with belimumab. While add-on belimumab overall induced increases in C3 and C4 levels, those could not distinguish patients who flared from patients who did not.

Among the limitations of the present study, one should mention the selected clinical trial population, which was enriched with patients with active musculoskeletal and mucocutaneous SLE, raising concerns about the generalisability of our findings. On the other hand, this is the first study to assess early changes in B cell subsets upon treatment initiation in relation to the development of SLE flares in a large study population. Importantly, when interpreting the results, one should bear in mind that we investigated relative and not absolute changes in cell subsets, which on the one hand may pose hurdles in interpretation and direct clinical implementation, whereas on the other hand normalised the values and circumvented batch effects from the varying methods at different laboratories. Lastly, in this investigation we stratified flares according to their severity, which forms a rather generalised concept for flaring. However, even severe articular or mucocutaneous flares may be less likely to result in life-threatening complications and irreversible organ damage compared with renal or neuropsychiatric flares. While it was beyond the scope of this study, flare stratification by organ involvement would have merit in a future analysis, as would stratification by background immunosuppressive therapy.

In summary, we showed that a rapid increase in long-lived plasma cells, an early increase in the total pool of circulating B cells, and an early or intermediate increase in memory B cells upon treatment initiation for active SLE heralded subsequent severe disease flares. Moreover, no or less prominent rapid or early decreases in anti-dsDNA antibody levels were also associated with the development of flares of any severity and severe flares, especially in patients treated with add-on belimumab. An initial expansion of memory B cells may signify sustained response to therapy when followed by a subsequent drop, while intermediate increases in memory B cells may portend flaring. Therapeutic adjustments in patients showing no dynamics in peripheral plasma cell subsets or anti-dsDNA levels might help prevent flares and disease progression. Overall, anti-dsDNA may be an important marker in the monitoring of patients treated with belimumab, and peripheral B cell and plasma cell subsets may prove a useful complement to traditional surveillance and early treatment evaluation in patients with SLE.

## Data Availability Statement

The original contributions presented in the study are included in the article/**Supplementary Material**. Further inquiries can be directed to the corresponding author.

## Ethics Statement

The trial protocols were approved by regional ethics review boards for all participating centres and complied with the ethical principles of the Declaration of Helsinki. Written informed consent was obtained from all study participants prior to enrolment. The present study was reviewed and approved by the Swedish Ethical Review Authority (2019-05498).

## Author Contributions

Study conception and design, IP and MG. Acquisition of data, IP, AG, JC, AB, and JL. Analysis and interpretation of data, IP, AG, and MG. All authors were involved in the drafting of the manuscript or revising it critically for important intellectual content, and all authors approved the final version to be submitted for publication.

## Funding

This work was supported by grants from the Swedish Rheumatism Association (R-941095), King Gustaf V’s 80-year Foundation (FAI-2020-0741), Professor Nanna Svartz Foundation (2020-00368), Ulla and Roland Gustafsson Foundation (2021-26), Region Stockholm (FoUI-955483) and Karolinska Institutet.

## Conflict of Interest

IP has received research funding and/or honoraria from Amgen, AstraZeneca, Aurinia Pharmaceuticals, Elli Lilly and Company, Gilead Sciences, GlaxoSmithKline, Janssen Pharmaceuticals, Novartis and F. Hoffmann-La Roche AG.

The remaining authors declare that the research was conducted in the absence of any commercial or financial relationships that could be construed as a potential conflict of interest.

## Publisher’s Note

All claims expressed in this article are solely those of the authors and do not necessarily represent those of their affiliated organizations, or those of the publisher, the editors and the reviewers. Any product that may be evaluated in this article, or claim that may be made by its manufacturer, is not guaranteed or endorsed by the publisher.

## References

[1] Doria A Iaccarino L Ghirardello A Zampieri S Arienti S Sarzi-Puttini P. Long-Term Prognosis and Causes of Death in Systemic Lupus Erythematosus. Am J Med (2006) 119(8):700–6. doi: 10.1016/j.amjmed.2005.11.034 16887417

[2] Bruce IN O'Keeffe AG Farewell V Hanly JG Manzi S Su L. Factors Associated With Damage Accrual in Patients With Systemic Lupus Erythematosus: Results From the Systemic Lupus International Collaborating Clinics (SLICC) Inception Cohort. Ann Rheum Dis (2015) 74(9):1706–13. doi: 10.1136/annrheumdis-2013-205171 PMC4552899 24834926

[3] Ugarte-Gil MF Acevedo-Vasquez E Alarcon GS Pastor-Asurza CA Alfaro-Lozano JL Cucho-Venegas JM. The Number of Flares Patients Experience Impacts on Damage Accrual in Systemic Lupus Erythematosus: Data From a Multiethnic Latin American Cohort. Ann Rheum Dis (2015) 74(6):1019–23. doi: 10.1136/annrheumdis-2013-204620 24525909

[4] Lee YH Choi SJ Ji JD Song GG. Overall and Cause-Specific Mortality in Systemic Lupus Erythematosus: An Updated Meta-Analysis. Lupus (2016) 25(7):727–34. doi: 10.1177/0961203315627202 26811368

[5] McElhone K Abbott J Hurley M Burnell J Lanyon P Rahman A. Flares in Patients With Systemic Lupus Erythematosus. Rheumatol (Oxford) (2021) 60(7):3262–7. doi: 10.1093/rheumatology/keaa777 PMC8517882 33325488

[6] Doria A Amoura Z Cervera R Khamastha MA Schneider M Richter J. Annual Direct Medical Cost of Active Systemic Lupus Erythematosus in Five European Countries. Ann Rheum Dis (2014) 73(1):154–60. doi: 10.1136/annrheumdis-2012-202443 23264339

[7] Gordon C Sutcliffe N Skan J Stoll T Isenberg DA. Definition and Treatment of Lupus Flares Measured by the BILAG Index. Rheumatol (Oxford) (2003) 42(11):1372–9. doi: 10.1093/rheumatology/keg382 12810926

[8] Isenberg DA Allen E Farewell V D'Cruz D Alarcon GS Aranow C. An Assessment of Disease Flare in Patients With Systemic Lupus Erythematosus: A Comparison of BILAG 2004 and the Flare Version of SELENA. Ann Rheum Dis (2011) 70(1):54–9. doi: 10.1136/ard.2010.132068 20833737

[9] Petri M. Disease Activity Assessment in SLE: Do We Have the Right Instruments? Ann Rheum Dis (2007) 66(Suppl 3):iii61–4. doi: 10.1136/ard.2007.078477 PMC2095289 17934099

[10] Petri M Buyon J Kim M. Classification and Definition of Major Flares in SLE Clinical Trials. Lupus (1999) 8(8):685–91. doi: 10.1191/096120399680411281 10568907

[11] Pisetsky DS Lipsky PE. New Insights Into the Role of Antinuclear Antibodies in Systemic Lupus Erythematosus. Nat Rev Rheumatol (2020) 16(10):565–79. doi: 10.1038/s41584-020-0480-7 PMC8456518 32884126

[12] Gensous N Marti A Barnetche T Blanco P Lazaro E Seneschal J. Predictive Biological Markers of Systemic Lupus Erythematosus Flares: A Systematic Literature Review. Arthritis Res Ther (2017) 19(1):238. doi: 10.1186/s13075-017-1442-6 29065901 PMC5655881

[13] Moroni G Quaglini S Radice A Trezzi B Raffiotta F Messa P. The Value of a Panel of Autoantibodies for Predicting the Activity of Lupus Nephritis at Time of Renal Biopsy. J Immunol Res (2015) 2015:106904. doi: 10.1155/2015/106904 25815344 PMC4357044

[14] Parodis I Stockfelt M Sjowall C. B Cell Therapy in Systemic Lupus Erythematosus: From Rationale to Clinical Practice. Front Med (Lausanne) (2020) 7:316. doi: 10.3389/fmed.2020.00316 32754605 PMC7381321

[15] Dooley MA Houssiau F Aranow C D'Cruz DP Askanase A Roth DA. Effect of Belimumab Treatment on Renal Outcomes: Results From the Phase 3 Belimumab Clinical Trials in Patients With SLE. Lupus (2013) 22(1):63–72. doi: 10.1177/0961203312465781 23263865

[16] Wallace DJ Ginzler EM Merrill JT Furie RA Stohl W Chatham WW. Safety and Efficacy of Belimumab Plus Standard Therapy for Up to Thirteen Years in Patients With Systemic Lupus Erythematosus. Arthritis Rheumatol (2019) 71(7):1125–34. doi: 10.1002/art.40861 PMC6617785 30771238

[17] Iaccarino L Bettio S Reggia R Zen M Frassi M Andreoli L. Effects of Belimumab on Flare Rate and Expected Damage Progression in Patients With Active Systemic Lupus Erythematosus. Arthritis Care Res (Hoboken) (2017) 69(1):115–23. doi: 10.1002/acr.22971 27390293

[18] Gatto M Saccon F Zen M Regola F Fredi M Andreoli L. Early Disease and Low Baseline Damage as Predictors of Response to Belimumab in Patients With Systemic Lupus Erythematosus in a Real-Life Setting. Arthritis Rheumatol (2020) 72(8):1314–24. doi: 10.1002/art.41253 32275125

[19] Parodis I Sjowall C Jonsen A Ramskold D Zickert A Frodlund M. Smoking and Pre-Existing Organ Damage Reduce the Efficacy of Belimumab in Systemic Lupus Erythematosus. Autoimmun Rev (2017) 16(4):343–51. doi: 10.1016/j.autrev.2017.02.005 28216072

[20] Navarra SV Guzman RM Gallacher AE Hall S Levy RA Jimenez RE. Efficacy and Safety of Belimumab in Patients With Active Systemic Lupus Erythematosus: A Randomised, Placebo-Controlled, Phase 3 Trial. Lancet (2011) 377(9767):721–31. doi: 10.1016/S0140-6736(10)61354-2 21296403

[21] Furie R Petri M Zamani O Cervera R Wallace DJ Tegzova D. Randomized, Placebo-Controlled Study of Belimumab, a Monoclonal Antibody That Inhibits B Lymphocyte Stimulator, in Patients With Systemic Lupus Erythematosus. Arthritis Rheum (2011) 63(12):3918–30. doi: 10.1002/art.30613 PMC5007058 22127708

[22] Parodis I Vital EM Hassan SU Jonsen A Bengtsson AA Eriksson P. *De Novo* Lupus Nephritis During Treatment With Belimumab. Rheumatol (Oxford) (2021) 60(9):4348–54. doi: 10.1093/rheumatology/keaa796 PMC8409994 33341888

[23] Stohl W Schwarting A Okada M Scheinberg M Doria A Hammer AE. Efficacy and Safety of Subcutaneous Belimumab in Systemic Lupus Erythematosus: A Fifty-Two-Week Randomized, Double-Blind, Placebo-Controlled Study. Arthritis Rheumatol (2017) 69(5):1016–27. doi: 10.1002/art.40049 PMC5434872 28118533

[24] Zhang F Bae SC Bass D Chu M Egginton S Gordon D. A Pivotal Phase III, Randomised, Placebo-Controlled Study of Belimumab in Patients With Systemic Lupus Erythematosus Located in China, Japan and South Korea. Ann Rheum Dis (2018) 77(3):355–63. doi: 10.1136/annrheumdis-2017-211631 PMC5867402 29295825

[25] Petri M Kim MY Kalunian KC Grossman J Hahn BH Sammaritano LR. Combined Oral Contraceptives in Women With Systemic Lupus Erythematosus. N Engl J Med (2005) 353(24):2550–8. doi: 10.1056/NEJMoa051135 16354891

[26] Furie RA Petri MA Wallace DJ Ginzler EM Merrill JT Stohl W. Novel Evidence-Based Systemic Lupus Erythematosus Responder Index. Arthritis Rheum (2009) 61(9):1143–51. doi: 10.1002/art.24698 PMC2748175 19714615

[27] Jacobi AM Odendahl M Reiter K Bruns A Burmester GR Radbruch A. Correlation Between Circulating CD27high Plasma Cells and Disease Activity in Patients With Systemic Lupus Erythematosus. Arthritis Rheum (2003) 48(5):1332–42. doi: 10.1002/art.10949 12746906

[28] Stohl W Hiepe F Latinis KM Thomas M Scheinberg MA Clarke A. Belimumab Reduces Autoantibodies, Normalizes Low Complement Levels, and Reduces Select B Cell Populations in Patients With Systemic Lupus Erythematosus. Arthritis Rheum (2012) 64(7):2328–37. doi: 10.1002/art.34400 PMC3350827 22275291

[29] Klasener K Jellusova J Andrieux G Salzer U Bohler C Steiner SN. CD20 as a Gatekeeper of the Resting State of Human B Cells. Proc Natl Acad Sci USA (2021) 118(7):e2021342118. doi: 10.1073/pnas.2021342118 PMC7896350 33563755

[30] Kaul A Gordon C Crow MK Touma Z Urowitz MB van Vollenhoven R. Systemic Lupus Erythematosus. Nat Rev Dis Primers (2016) 2:16039. doi: 10.1038/nrdp.2016.39 27306639

[31] Gatto M Zen M Iaccarino L Doria A. New Therapeutic Strategies in Systemic Lupus Erythematosus Management. Nat Rev Rheumatol (2019) 15(1):30–48. doi: 10.1038/s41584-018-0133-2 30538302

[32] Zen M Bassi N Nalotto L Canova M Bettio S Gatto M. Disease Activity Patterns in a Monocentric Cohort of SLE Patients: A Seven-Year Follow-Up Study. Clin Exp Rheumatol (2012) 30(6):856–63. 22765883

[33] Gyori N Giannakou I Chatzidionysiou K Magder L van Vollenhoven RF Petri M. Disease Activity Patterns Over Time in Patients With SLE: Analysis of the Hopkins Lupus Cohort. Lupus Sci Med (2017) 4(1):e000192. doi: 10.1136/lupus-2016-000192 28243457 PMC5307372

[34] Ramskold D Parodis I Lakshmikanth T Sippl N Khademi M Chen Y. B Cell Alterations During BAFF Inhibition With Belimumab in SLE. EBioMedicine (2019) 40:517–27. doi: 10.1016/j.ebiom.2018.12.035 PMC6412067 30593436

[35] Regola F Piantoni S Lowin T Archetti S Reggia R Kumar R. Association Between Changes in BLyS Levels and the Composition of B and T Cell Compartments in Patients With Refractory Systemic Lupus Erythematosus Treated With Belimumab. Front Pharmacol (2019) 10:433. doi: 10.3389/fphar.2019.00433 31105569 PMC6494924

[36] Jacobi AM Huang W Wang T Freimuth W Sanz I Furie R. Effect of Long-Term Belimumab Treatment on B Cells in Systemic Lupus Erythematosus: Extension of a Phase II, Double-Blind, Placebo-Controlled, Dose-Ranging Study. Arthritis Rheum (2010) 62(1):201–10. doi: 10.1002/art.27189 PMC2857977 20039404

[37] Vital EM Dass S Buch MH Henshaw K Pease CT Martin MF. B Cell Biomarkers of Rituximab Responses in Systemic Lupus Erythematosus. Arthritis Rheum (2011) 63(10):3038–47. doi: 10.1002/art.30466 21618204

[38] Md Yusof MY Shaw D El-Sherbiny YM Dunn E Rawstron AC Emery P. Predicting and Managing Primary and Secondary Non-Response to Rituximab Using B-Cell Biomarkers in Systemic Lupus Erythematosus. Ann Rheum Dis (2017) 76(11):1829–36. doi: 10.1136/annrheumdis-2017-211191 PMC5705851 28684557

[39] Parodis I Johansson P Gomez A Soukka S Emamikia S Chatzidionysiou K. Predictors of Low Disease Activity and Clinical Remission Following Belimumab Treatment in Systemic Lupus Erythematosus. Rheumatol (Oxford) (2019) 58(12):2170–6. doi: 10.1093/rheumatology/kez191 PMC6880848 31157891

[40] van Vollenhoven RF Petri MA Cervera R Roth DA Ji BN Kleoudis CS. Belimumab in the Treatment of Systemic Lupus Erythematosus: High Disease Activity Predictors of Response. Ann Rheum Dis (2012) 71(8):1343–9. doi: 10.1136/annrheumdis-2011-200937 PMC3396451 22337213

[41] Parodis I Akerstrom E Sjowall C Sohrabian A Jonsen A Gomez A. Autoantibody and Cytokine Profiles During Treatment With Belimumab in Patients With Systemic Lupus Erythematosus. Int J Mol Sci (2020) 21(10):3463. doi: 10.3390/ijms21103463 PMC7278961 32422945

[42] Furie RA Aroca G Cascino MD Garg JP Rovin BH Alvarez A. B-Cell Depletion With Obinutuzumab for the Treatment of Proliferative Lupus Nephritis: A Randomised, Double-Blind, Placebo-Controlled Trial. Ann Rheumatic Dis (2022) 81(1):100-7. doi: 10.1136/annrheumdis-2021-220920 PMC8762029 34615636

[43] Pisetsky DS. Anti-DNA Antibodies–Quintessential Biomarkers of SLE. Nat Rev Rheumatol (2016) 12(2):102–10. doi: 10.1038/nrrheum.2015.151 26581343

[44] Bragazzi NL Watad A Damiani G Adawi M Amital H Shoenfeld Y. Role of Anti-DNA Auto-Antibodies as Biomarkers of Response to Treatment in Systemic Lupus Erythematosus Patients: Hypes and Hopes. Insights and Implications From a Comprehensive Review of the Literature. Expert Rev Mol Diagn (2019) 19(11):969–78. doi: 10.1080/14737159.2019.1665511 31516059

[45] Golder V Kandane-Rathnayake R Huq M Louthrenoo W Luo SF Wu Y-JJ. Evaluation of Remission Definitions for Systemic Lupus Erythematosus: A Prospective Cohort Study. Lancet Rheumatol (2019) 1(2):e103–e10. doi: 10.1016/S2665-9913(19)30048-7 38229337

