# Supplementary material for: Early B Cell and Plasma Cell Kinetics Upon Treatment Initiation Portend Flares in Systemic Lupus Erythematosus: A Post-Hoc Analysis of Three Phase III Clinical Trials of Belimumab
Source: Front Immunol. 2022 Apr 4;13:796508. doi: 10.3389/fimmu.2022.796508 (PMC9015703; doi:10.3389/fimmu.2022.796508)
Supplement: Supplementary file 1 [file Table_1.pdf]

Supplementary Table S1. Relative to baseline percentage change in B cell subset counts and serum levels of serological markers from baseline through week 8 in patients who developed versus did not develop flares from week 24 through week 52 in the pooled BLISS study population.

	**Any flare**			**Severe flare**		
	**Yes**	**No**	**P value**	**Yes**	**No**	**P value**
**Entire patient cohort** (all treatment arms)						
**B cell subsets**						
$\mathbf{CD19^{+}CD20^{+}}$	-4.1 (-30.2−36.7)	0.0 (-29.9−48.9)	0.211	-5.6 (-30.2−45.1)	-2.0 (-30.0−40.9)	0.969
$\mathbf{CD19^{+}CD20^{+}CD27^{+}}$	76.7 (0.0−188.9)	84.6 (6.1−200.0)	0.179	50.0 (-16.7−200.0)	83.5 (6.7−192.2)	**0.037**
$\mathbf{CD19^{+}CD20^{+}CD69^{+}}$	-17.3 (-64.6−110.7)	-10.5 (-68.7−96.0)	0.561	-11.5 (-61.2−134.9)	-16.2 (-66.7−105.7)	0.378
$\mathbf{CD19^{+}CD20^{+}CD27^{-}}$	-17.5 (-44.6−21.1)	-15.5 (-44.3−17.9)	0.633	-12.4 (-41.3−27.1)	-17.0 (-44.6−17.9)	0.273
$\mathbf{CD19^{+}CD20^{+}CD138^{+}}$	-20.6 (-67.8−80.0)	-12.0 (-69.8−114.0)	0.496	-4.0 (-68.0−113.1)	-18.6 (-68.3−97.1)	0.599
$\mathbf{CD19^{+}CD20^{-}CD138^{+}}$	-19.5 (-65.4−56.0)	-28.0 (-68.5−63.5)	0.194	-11.9 (-58.7−81.1)	-24.0 (-67.7−54.1)	0.123
$\mathbf{CD19^{+}CD20^{-}CD27^{brt}}$	-30.6 (-66.7−33.3)	-30.0 (-68.4−7.5)	0.240	-21.8 (-62.8−93.8)	-31.1 (-66.7−17.8)	0.105
$\mathbf{CD19^{+}CD27^{brt}CD38^{brt}}$	-22.8 (-62.5−58.1)	-29.1 (-61.8−37.7)	0.354	-24.8 (-62.9−58.5)	-26.4 (-61.8−49.3)	0.967
**Serological markers**						
**C3**	2.0 (-9.0−14.5)	2.7 (-7.5−15.5)	0.247	0.5 (-8.5−14.6)	2.7 (-8.4−15.1)	0.662
**C4**	6.3 (-7.3−23.6)	8.3 (-5.9−28.6)	0.080	4.9 (-11.5−25.0)	7.1 (-6.3−25.0)	0.235
**anti-dsDNA** (all patients)	0.0 (-27.3−0.2)	-12.5 (-34.1−0.0)	**<0.001**	-4.7 (-27.1−1.8)	-4.9 (-31.2−0.0)	0.339
**anti-dsDNA** (patients positive at baseline)	-15.8 (-36.5−8.1)	-22.2 (-39.9−-0.6)	**0.001**	-14.2 (-36.5−7.3)	-19.1 (-38.6−3.8)	0.193
**Belimumab**						
**B cell subsets**						
$\mathbf{CD19^{+}CD20^{+}}$	-4.8 (-35.3−43.9)	0.0 (-31.6−60.0)	0.243	-7.1 (-37.7−63.1)	-2.1 (-32.3−50.6)	0.971
$\mathbf{CD19^{+}CD20^{+}CD27^{+}}$	128.6 (61.1−250.0)	126.3 (50.0−250.0)	0.548	114.3 (-38.9−281.7)	128.6 (58.3−242.9)	0.620
$\mathbf{CD19^{+}CD20^{+}CD69^{+}}$	-19.0 (-64.3−107.2)	-10.5 (-67.4−96.0)	0.874	-10.8 (-63.6−136.7)	-17.9 (-66.5−100.8)	0.447
$\mathbf{CD19^{+}CD20^{+}CD27^{-}}$	-29.9 (-53.0−12.8)	-25.3 (-49.6−14.2)	0.157	-20.8 (-53.8−22.6)	-28.3 (-51.3−13.1)	0.765
$\mathbf{CD19^{+}CD20^{+}CD138^{+}}$	-19.2 (-67.4−80.2)	-11.3 (-68.2−124.4)	0.691	-0.2 (-63.7−118.4)	-16.6 (-68.1−100.0)	0.296
$\mathbf{CD19^{+}CD20^{-}CD138^{+}}$	-23.8 (-69.0−46.1)	-33.3 (-71.3−63.6)	0.449	-23.6 (-66.3−74.4)	-28.9 (-70.8−50.8)	0.567
$\mathbf{CD19^{+}CD20^{-}CD27^{brt}}$	-36.4 (-71.1−14.3)	-40.0 (-75.0−0.0)	0.402	-39.2 (-66.7−40.7)	-37.5 (-72.7−0.0)	0.393
$\mathbf{CD19^{+}CD27^{brt}CD38^{brt}}$	-31.9 (-65.7−39.9)	-37.0 (-65.4−22.8)	0.384	-42.9 (-73.2−25.3)	-33.7 (-65.1−34.3)	0.385
**Serological markers**						
**C3**	3.3 (-7.9−15.2)	5.1 (-6.4−18.7)	0.091	2.2 (-7.4−15.1)	3.8 (-6.8−16.4)	0.497
**C4**	9.1 (-3.6−28.2)	11.8 (-4.4−32.2)	0.264	9.1 (-3.3−30.8)	10.0 (-4.0−29.4)	0.838
**anti-dsDNA** (all patients)	-4.6 (-30.7−0.0)	-17.7 (-37.8−0.0)	**<0.001**	-8.7 (-31.3−1.3)	-12.3 (-34.5−0.0)	0.302
**anti-dsDNA** (patients positive at baseline)	-20.2 (-42.5−2.7)	-27.4 (-43.2−-6.3)	**0.012**	-16.5 (-43.9−5.9)	-23.8 (-43.0−-2.9)	0.165
**Placebo**						
**B cell subsets**						
$\mathbf{CD19^{+}CD20^{+}}$	-3.1 (-24.4−31.9)	0.0 (-25.9−26.5)	0.630	-3.5 (-24.4−34.1)	-2.0 (-24.9−27.8)	0.972
$\mathbf{CD19^{+}CD20^{+}CD27^{+}}$	-2.1 (-34.7−33.3)	4.2 (-25.0−39.7)	**0.037**	-11.1 (-47.2−47.1)	0.0 (-28.6−35.7)	0.399
$\mathbf{CD19^{+}CD20^{+}CD69^{+}}$	-14.8 (-64.8−127.3)	-12.6 (-69.9−96.6)	0.439	-11.5 (-60.6−130.0)	-15.3 (-68.0−116.4)	0.653
$\mathbf{CD19^{+}CD20^{+}CD27^{-}}$	-2.5 (-24.0−32.7)	-2.4 (-26.3−26.8)	0.924	0.0 (-25.0−33.3)	-2.6 (-25.3−31.6)	0.761
$\mathbf{CD19^{+}CD20^{+}CD138^{+}}$	-23.4 (-67.9−72.1)	-13.6 (-72.2−110.3)	0.562	-9.3 (-73.4−88.6)	-19.5 (-69.0−89.2)	0.755
$\mathbf{CD19^{+}CD20^{-}CD138^{+}}$	-11.3 (-58.9−68.3)	-12.4 (-62.6−63.6)	0.400	2.4 (-47.0−86.2)	-14.2 (-62.1−64.9)	0.144
$\mathbf{CD19^{+}CD20^{-}CD27^{brt}}$	-14.4 (-53.2−66.7)	0.0 (-50.0−51.6)	0.967	-2.7 (-54.0−115.4)	-7.6 (-50.0−51.1)	0.272
$\mathbf{CD19^{+}CD27^{brt}CD38^{brt}}$	-2.9 (-54.1−86.1)	-0.9 (-48.3−73.8)	0.681	-5.4 (-50.9−93.3)	-1.4 (-51.3−77.1)	0.830
**Serological markers**						
**C3**	0.0 (-10.2−13.1)	-0.9 (-10.0−7.9)	0.451	0.0 (-8.9−12.8)	-0.7 (-10.2−10.1)	0.591
**C4**	0.0 (-14.3−16.7)	3.3 (-10.0−15.4)	0.413	0.0 (-15.8−16.7)	0.0 (-12.5−16.7)	0.434
**anti-dsDNA** (all patients)	0.0 (-21.5−9.2)	0.0 (-23.6−12.6)	0.750	0.0 (-24.7−9.8)	0.0 (-21.6−10.8)	0.569
**anti-dsDNA** (patients positive at baseline)	-4.0 (-28.2−17.9)	-9.4 (-29.6−15.1)	0.439	-8.7 (-29.8−15.2)	-4.4 (-28.2−-17.6)	0.544

Data are presented as medians (interquartile range) of the relative to baseline percentage changes. P values are derived from non-parametrical Mann-Whitney *U* tests. Statistically significant P values are in bold.

C3: complement component 3; C4: complement component 4
