# Supplementary material for: Early B Cell and Plasma Cell Kinetics Upon Treatment Initiation Portend Flares in Systemic Lupus Erythematosus: A Post-Hoc Analysis of Three Phase III Clinical Trials of Belimumab
Source: Front Immunol. 2022 Apr 4;13:796508. doi: 10.3389/fimmu.2022.796508 (PMC9015703; doi:10.3389/fimmu.2022.796508)
Supplement: Supplementary file 2 [file Table_2.pdf]

Supplementary Table S2. Relative to baseline percentage change in B cell subset counts and serum levels of serological markers from baseline through week 24 in patients who developed versus did not develop flares from week 24 through week 52 in the pooled BLISS study population.

	**Any flare**			**Severe flare**		
	**Yes**	**No**	**P value**	**Yes**	**No**	**P value**
**Entire patient cohort** (all treatment arms)						
**B cell subsets**						
$\mathbf{CD19^{+}CD20^{+}}$	-18.6 (-48.7−21.3)	-21.4 (-51.9−23.5)	0.575	-14.3 (-53.8−29.5)	-21.0 (-50.0−20.6)	0.323
$\mathbf{CD19^{+}CD20^{+}CD27^{+}}$	53.8 (-3.8−141.7)	56.5 (-10.8−151.3)	0.863	52.2 (-4.3−141.7)	54.5 (-9.1−150.0)	0.691
$\mathbf{CD19^{+}CD20^{+}CD69^{+}}$	-32.6 (-72.9−75.7)	-28.6 (-72.5−86.9)	0.532	-21.8 (-70.0−107.3)	-31.4 (-73.1−75.0)	0.431
$\mathbf{CD19^{+}CD20^{+}CD27^{-}}$	-36.8 (-65.0−2.5)	-42.1 (-66.3−-1.1)	0.400	-37.5 (-66.7−12.9)	-38.9 (-65.2−0.0)	0.313
$\mathbf{CD19^{+}CD20^{+}CD138^{+}}$	-40.0 (-74.9−52.8)	-40.4 (-80.0−73.0)	0.727	-21.5 (-68.5−108.7)	-41.1 (-78.1−60.9)	**0.024**
$\mathbf{CD19^{+}CD20^{-}CD138^{+}}$	-37.0 (-73.8−41.4)	-39.9 (-78.6−39.3)	0.331	-23.5 (-67.2−51.0)	-39.4 (-77.0−38.6)	**0.028**
$\mathbf{CD19^{+}CD20^{-}CD27^{brt}}$	-37.6 (-73.3−24.0)	-33.9 (-71.5−0.0)	0.603	-28.7 (-67.8−74.8)	-37.7 (-73.7−10.7)	0.088
$\mathbf{CD19^{+}CD27^{brt}CD38^{brt}}$	-24.1 (-66.2−59.2)	-30.1 (-65.8−23.6)	0.071	-19.0 (-62.5−113.6)	-27.8 (-66.2−39.2)	**0.045**
**Serological markers**						
**C3**	2.3 (-7.8−15.9)	2.5 (-8.2−16.5)	0.946	1.0 (-10.0−17.4)	2.6 (-7.9−16.0)	0.891
**C4**	10.5 (-7.1−33.6)	11.8 (-3.8−33.3)	0.310	12.5 (-8.3−37.5)	11.1 (-5.9−33.3)	0.991
**anti-dsDNA** (all patients)	-1.9 (-39.1−0.0)	-21.7 (-47.9−0.0)	**<0.001**	0.0 (-36.5−9.7)	-13.3 (-44.0−0.0)	**0.020**
**anti-dsDNA** (patients positive at baseline)	-24.0 (-48.6−7.4)	-33.0 (-54.6−-6.5)	**<0.001**	-11.2 (-49.5−17.1)	-29.8 (-51.6−-2.4)	**0.003**
**Belimumab**						
**B cell subsets**						
$\mathbf{CD19^{+}CD20^{+}}$	-31.8 (-59.1−9.6)	-30.6 (-57.3−22.5)	0.288	-36.3 (-61.9−13.1)	-31.2 (-57.6−14.6)	0.421
$\mathbf{CD19^{+}CD20^{+}CD27^{+}}$	93.8 (26.3−184.2)	86.6 (16.7−200.0)	0.597	83.3 (26.0−163.9)	90.0 (20.0−197.1)	0.454
$\mathbf{CD19^{+}CD20^{+}CD69^{+}}$	-36.6 (-73.7−67.5)	-27.4 (-71.6−90.3)	0.269	-39.1 (-74.9−104.1)	-32.9 (-73.2−75.1)	0.815
$\mathbf{CD19^{+}CD20^{+}CD27^{-}}$	-55.1 (-73.4−28.6)	-53.8 (-70.6−18.8)	0.172	-58.3 (-74.1−35.9)	-54.1 (-72.2−-23.0)	0.225
$\mathbf{CD19^{+}CD20^{+}CD138^{+}}$	-47.6 (-78.1−34.9)	-42.9 (-82.0−59.8)	0.519	-41.2 (-70.7−68.1)	-46.9 (-80.4−44.4)	0.217
$\mathbf{CD19^{+}CD20^{-}CD138^{+}}$	-46.6 (-78.9−33.3)	-43.0 (-78.5−35.8)	0.381	-48.3 (-75.6−27.2)	-43.5 (-79.0−34.5)	0.967
$\mathbf{CD19^{+}CD20^{-}CD27^{brt}}$	-50.0 (-77.7−8.6)	-48.4 (-75.2−0.0)	0.899	-43.6 (-76.1−43.2)	-50.0 (-77.0−0.0)	0.468
$\mathbf{CD19^{+}CD27^{brt}CD38^{brt}}$	-38.3 (-70.7−43.8)	-38.3 (-68.2−3.9)	0.389	-37.5 (-73.1−105.6)	-46.2 (-76.0−22.3)	0.878
**Serological markers**						
**C3**	4.0 (-6.0−16.8)	4.6 (-7.3−18.5)	0.913	2.2 (-6.8−17.6)	4.4 (-6.5−17.7)	0.987
**C4**	14.3 (0.0−40.0)	15.2 (0.0−40.0)	0.670	16.3 (0.0−55.4)	14.3 (0.0−40.0)	0.248
**anti-dsDNA** (all patients)	-10.8 (-45.0−0.0)	-26.4 (-52.0−0.0)	**<0.001**	-10.5 (-43.7−8.2)	-20.5 (-48.3−0.0)	0.071
**anti-dsDNA** (patients positive at baseline)	-31.5 (-52.5−-3.3)	-39.0 (-56.9−14.7)	**0.008**	-19.6 (-57.9−13.4)	-35.9 (-54.3−-10.4)	**0.022**
**Placebo**						
**B cell subsets**						
$\mathbf{CD19^{+}CD20^{+}}$	0.7 (-27.3−33.9)	-5.2 (-39.3−24.6)	0.082	11.7 (-23.6−50.6)	-2.8 (-31.0−30.7)	0.062
$\mathbf{CD19^{+}CD20^{+}CD27^{+}}$	0.0 (-33.3−54.4)	-11.6 (-38.6−47.5)	**0.038**	14.3 (-26.1−84.3)	-7.7 (-36.4−50.0)	**0.023**
$\mathbf{CD19^{+}CD20^{+}CD69^{+}}$	-25.0 (-69.2−81.8)	-30.3 (-75.7−71.2)	0.681	3.2 (-64.3−112.8)	-30.4 (-72.3−74.2)	0.159
$\mathbf{CD19^{+}CD20^{+}CD27^{-}}$	0.0 (-26.4−38.6)	-5.7 (-38.3−27.6)	0.104	4.8 (-19.3−57.3)	-3.9 (-31.7−31.6)	0.101
$\mathbf{CD19^{+}CD20^{+}CD138^{+}}$	-15.8 (-66.3−93.8)	-31.7 (-76.6−81.5)	0.270	-6.7 (-54.9−140.1)	-24.0 (-74.0−81.5)	0.106
$\mathbf{CD19^{+}CD20^{-}CD138^{+}}$	-16.1 (-60.1−63.6)	-35.1 (-78.7−59.0)	**0.012**	6.7 (-39.5−143.7)	-27.2 (-70.7−52.3)	**0.002**
$\mathbf{CD19^{+}CD20^{-}CD27^{brt}}$	-14.8 (-55.2−60.0)	-7.4 (-52.0−38.4)	0.772	-0.7 (-52.6−85.8)	-14.3 (-53.9−46.3)	0.184
$\mathbf{CD19^{+}CD27^{brt}CD38^{brt}}$	4.3 (-48.4−85.2)	-8.5 (-52.5−96.2)	0.366	41.2 (-37.2−154.6)	-6.1 (-50.8−81.2)	**0.038**
**Serological markers**						
**C3**	-1.2 (-12.2−14.3)	-1.1 (-10.1−9.0)	0.497	-1.9 (-14.2−17.4)	-1.1 (-10.7−10.7)	0.845
**C4**	0.0 (-14.3−23.5)	3.3 (-12.0−18.8)	0.802	0.0 (-20.0−25.0)	0.0 (12.5−20.0)	0.723
**anti-dsDNA** (all patients)	0.0 (-25.1−12.5)	-3.4 (-33.3−5.6)	0.087	0.0 (-24.3−22.5)	0.0 (-30.2−10.3)	0.525
**anti-dsDNA** (patients positive at baseline)	-7.9 (-35.4−21.8)	-21.2 (-42.4−11.4)	0.106	-4.9 (-36.5−24.6)	-16.7 (-37.2−16.9)	0.316

Data are presented as medians (interquartile range) of the relative to baseline percentage changes. P values are derived from non-parametrical Mann-Whitney *U* tests. Statistically significant P values are in bold.

C3: complement component 3; C4: complement component 4
