# Supplementary material for: Early B Cell and Plasma Cell Kinetics Upon Treatment Initiation Portend Flares in Systemic Lupus Erythematosus: A Post-Hoc Analysis of Three Phase III Clinical Trials of Belimumab
Source: Front Immunol. 2022 Apr 4;13:796508. doi: 10.3389/fimmu.2022.796508 (PMC9015703; doi:10.3389/fimmu.2022.796508)
Supplement: Supplementary file 3 [file Table_3.pdf]

Supplementary Table S3. Relative to baseline percentage change in B cell subset counts and serum levels of serological markers from baseline through week 52 in patients who developed versus did not develop flares from week 24 through week 52 in the pooled BLISS study population.

	**Any flare**			**Severe flare**		
	**Yes**	**No**	**P value**	**Yes**	**No**	**P value**
**Entire patient cohort** (all treatment arms)						
**B cell subsets**						
**CD19^{+}CD20^{+}**	-36.6 (-65.2−1.4)	-42.9 (-66.7−-2.9)	0.228	-35.4 (-53.2−25.6)	-40.0 (-66.5−-1.1)	0.084
**CD19^{+}CD20^{+}CD27^{+}**	20.0 (-28.6−100.0)	13.8 (-27.8−87.8)	0.336	23.0 (-29.0−97.4)	15.4 (-28.6−94.3)	0.910
**CD19^{+}CD20^{+}CD69^{+}**	-42.9 (-78.8−34.3)	-37.2 (-79.5−55.9)	0.521	-59.8 (-81.8−31.8)	-40.0 (-79.3−43.8)	0.439
**CD19^{+}CD20^{+}CD27^{-}**	-54.1 (-77.7−-12.1)	-59.7 (-77.7−-17.7)	0.208	-48.5 (-73.1−1.2)	-56.3 (-77.9−14.3)	0.103
**CD19^{+}CD20^{+}CD138^{+}**	-45.8 (-81.8−35.2)	-54.1 (-81.8−23.6)	0.233	-47.8 (-85.5−32.7)	-49.7 (-81.7−31.3)	0.897
**CD19^{+}CD20^{-}CD138^{+}**	-41.7 (-76.1−42.2)	-49.3 (-82.0−37.5)	0.060	-21.5 (-75.3−83.1)	-46.1 (-78.6−38.1)	0.083
**CD19^{+}CD20^{-}CD27^{brt}**	-33.3 (-75.0−27.5)	-47.7 (-80.0−0.0)	**0.042**	-21.7 (-72.0−67.7)	-40.0 (-77.6−12.1)	0.110
**CD19^{+}CD27^{brt}CD38^{brt}**	-34.4 (-69.8−42.9)	-36.2 (-72.7−34.3)	0.252	-21.4 (-72.6−65.8)	-35.2 (-71.5−39.0)	0.592
**Serological markers**						
**C3**	3.7 (-8.3−19.5)	4.5 (-6.6−17.3)	0.080	3.9 (-7.1−21.0)	4.3 (-7.7−18.4)	0.923
**C4**	14.3 (-4.9−40.0)	15.8 (0.0−42.9)	0.153	16.7 (-4.5−51.0)	14.3 (-3.4−41.2)	0.882
**anti-dsDNA** (all patients)	-12.5 (-50.4−0.0)	-30.9 (-62.2−0.0)	**<0.001**	0.0 (-47.5−9.4)	-24.7 (-56.4−0.0)	**0.013**
**anti-dsDNA** (patients positive at baseline)	-37.9 (-62.6−0.1)	-46.0 (-67.0−-17.8)	**0.001**	-20.6 (-61.8−33.4)	-42.8 (-65.1−10.9)	**0.040**
**Belimumab**						
**B cell subsets**						
**CD19^{+}CD20^{+}**	-51.0 (-72.5−19.9)	-53.7 (-71.4−14.5)	0.864	-47.7 (-66.1−-21.9)	-53.4 (-72.2−-17.8)	0.378
**CD19^{+}CD20^{+}CD27^{+}**	35.7 (-14.3−123.1)	28.6 (-14.3−109.3)	0.399	38.2 (-27.2−116.4)	33.3 (-14.2−115.8)	0.649
**CD19^{+}CD20^{+}CD69^{+}**	-46.8 (-81.8−31.5)	-40.9 (-81.2−50.9)	0.538	-69.4 (-89.7−-26.2)	-42.5 (-81.0−40.9)	0.053
**CD19^{+}CD20^{+}CD27^{-}**	-69.9 (-83.1−-48.1)	-69.2 (-82.1−47.9)	0.686	-67.9 (-79.2−-48.2)	-69.5 (-82.6−-48.0)	0.630
**CD19^{+}CD20^{+}CD138^{+}**	-56.9 (-85.7−7.4)	-56.1 (-81.3−13.2)	0.607	-75.6 (-90.8−37.5)	-55.5 (-83.0−13.2)	**0.046**
**CD19^{+}CD20^{-}CD138^{+}**	-46.2 (-79.0−27.5)	-52.9 (-83.2−17.6)	0.291	-33.2 (-77.7−77.7)	-49.9 (-80.5−20.2)	0.271
**CD19^{+}CD20^{-}CD27^{brt}**	-45.0 (-82.7−13.8)	-53.0 (-87.1−0.0)	0.075	-26.3 (-72.8−44.0)	-50.0 (-83.7−0.0)	0.231
**CD19^{+}CD27^{brt}CD38^{brt}**	-44.5 (-76.3−26.6)	-48.1 (-75.8−13.8)	0.501	-40.4 (-74.3−16.9)	-46.2 (-76.0−22.3)	0.998
**Serological markers**						
**C3**	-6.9 (-6.4−22.8)	6.1 (-3.7−22.4)	0.443	10.2 (-6.6−29.4)	6.5 (-4.7−22.6)	0.939
**C4**	18.8 (0.0−46.2)	22.2 (4.5−50.0)	0.142	31.3 (8.0−58.1)	20.0 (0.0−47.6)	0.145
**anti-dsDNA** (all patients)	-26.0 (-56.5−0.0)	-37.5 (-65.1−0.0)	**<0.001**	-6.4 (-56.0−0.0)	-31.3 (-61.9−0.0)	0.149
**anti-dsDNA** (patients positive at baseline)	-46.2 (-66.2−-16.4)	-48.3 (-68.5−-21.3)	0.123	-51.3 (-75.6−46.7)	-47.5 (-67.2−-20.8)	0.684
**Placebo**						
**B cell subsets**						
**CD19^{+}CD20^{+}**	-7.3 (-39.0−32.5)	-14.3 (-42.4−29.8)	0.133	8.5 (-42.3−84.4)	-10.5 (-39.7−30.8)	0.237
**CD19^{+}CD20^{+}CD27^{+}**	-7.7 (-38.4−55.6)	-18.6 (-50.0−33.3)	0.105	-6.4 (-45.5−79.7)	13.8 (-44.7−50.0)	0.353
**CD19^{+}CD20^{+}CD69^{+}**	-34.5 (-71.2−41.9)	-18.8 (-74.6−80.3)	0.612	13.9 (-61.7−114.9)	-34.1 (-73.0−53.7)	0.292
**CD19^{+}CD20^{+}CD27^{-}**	-5.9 (-37.6−35.1)	-16.2 (-42.1−27.2)	0.086	14.9 (-41.6−82.7)	-11.0 (-40.2−30.8)	0.135
**CD19^{+}CD20^{+}CD138^{+}**	-7.8 (-73.6−116.0)	-39.8 (-84.1−48.3)	**0.017**	15.5 (-41.9−74.2)	-30.1 (-78.6−76.4)	0.131
**CD19^{+}CD20^{-}CD138^{+}**	-30.2 (-65.0−82.9)	-36.8 (-77.0−75.0)	0.176	7.5 (-74.1−132.3)	-34.5 (-70.3−75.0)	0.282
**CD19^{+}CD20^{-}CD27^{brt}**	-15.6 (-57.1−91.0)	-20.0 (-54.0−40.8)	0.607	0.3 (-68.5−232.2)	-18.2 (-54.6−51.3)	0.414
**CD19^{+}CD27^{brt}CD38^{brt}**	-11.1 (-54.4−100.6)	-14.9 (-57.5−59.8)	0.638	12.1 (-53.5−120.5)	-13.9 (-55.7−80.9)	0.583
**Serological markers**						
**C3**	-0.7 (-12.5−13.3)	-2.2 (-11.7−9.0)	0.276	-1.4 (-9.5−19.7)	-1.3 (-12.0−11.1)	0.634
**C4**	0.0 (-15.8−23.1)	0.0 (-12.5−20.0)	0.763	0.0 (-25.4−21.1)	0.0 (-14.3−21.4)	0.411
**anti-dsDNA** (all patients)	0.0 (-36.6−11.9)	-14.8 (-47.5−0.0)	**0.005**	0.0 (-23.2−19.6)	-1.0 (-42.8−5.3)	0.206
**anti-dsDNA** (patients positive at baseline)	-17.3 (-48.1−23.4)	-35.6 (-53.7−-1.6)	**0.006**	-3.8 (-37.8−31.8)	-24.7 (-51.6−10.2)	0.135

Data are presented as medians (interquartile range) of the relative to baseline percentage changes. P values are derived from non-parametrical Mann-Whitney *U* tests. Statistically significant P values are in bold.

C3: complement component 3; C4: complement component 4
