# Supplementary material for: Early B Cell and Plasma Cell Kinetics Upon Treatment Initiation Portend Flares in Systemic Lupus Erythematosus: A Post-Hoc Analysis of Three Phase III Clinical Trials of Belimumab
Source: Front Immunol. 2022 Apr 4;13:796508. doi: 10.3389/fimmu.2022.796508 (PMC9015703; doi:10.3389/fimmu.2022.796508)
Supplement: Supplementary file 4 [file Table_4.pdf]

Supplementary Table S4. Results from Cox proportional hazards regression, with any or severe flare from week 24 through week 76 as the outcome and relative to baseline B cell % change through week 8, 24 or 24 adjusted for change from baseline through week 8 as the exposure in the pooled BLISS study population.

	HR	95% CI	P value	HR	95% CI	P value
	Any flare			Severe flare		
**Baseline through week 8**						
**CD19^{+}CD20^{+}**	1.01	0.89−1.16	0.857	0.92	0.69−1.25	0.604
**Belimumab use**	0.78	0.64−0.94	**0.009**	0.67	0.46−0.99	**0.046**
**Interaction**	1.01	0.75−1.37	0.943	0.93	0.49−1.78	0.833
**CD19^{+}CD20^{+}CD27^{+}**	1.00	0.94−1.06	0.989	0.90	0.77−1.06	0.212
**Belimumab use**	0.78	0.64−0.96	**0.020**	0.77	0.50−1.18	0.224
**Interaction**	1.01	0.85−1.20	0.917	1.14	0.74−1.77	0.545
**CD19^{+}CD20^{+}CD69^{+}**	1.01	0.98−1.04	0.703	0.99	0.92−1.07	0.847
**Belimumab use**	0.76	0.63−0.92	**0.005**	0.65	0.44−0.97	**0.033**
**Interaction**	1.01	0.95−1.08	0.727	1.02	0.89−1.18	0.773
**CD19^{+}CD20^{+}CD27^{-}**	1.01	0.86−1.18	0.924	0.89	0.62−1.27	0.528
**Belimumab use**	0.78	0.64−0.95	**0.012**	0.66	0.45−0.97	**0.036**
**Interaction**	0.94	0.66−1.33	0.708	0.91	0.43−1.93	0.808
**CD19^{+}CD20^{+}CD138^{+}**	1.01	0.97−1.05	0.526	0.95	0.86−1.05	0.338
**Belimumab use**	0.78	0.65−0.95	**0.011**	0.67	0.45−0.99	**0.045**
**Interaction**	0.98	0.90−1.07	0.665	1.03	0.84−1.27	0.743
**CD19^{+}CD20^{-}CD138^{+}**	1.05	0.99−1.10	0.079	1.11	1.01−1.22	**0.024**
**Belimumab use**	0.79	0.65−0.95	**0.014**	0.69	0.47−1.03	0.066
**Interaction**	1.02	0.91−1.14	0.708	1.05	0.86−1.28	0.614
**CD19^{+}CD20^{-}CD27^{brt}**	1.03	0.98−1.09	0.248	1.08	0.98−1.20	0.119
**Belimumab use**	0.77	0.63−0.93	**0.006**	0.66	0.45−0.97	**0.036**
**Interaction**	0.94	0.83−1.06	0.303	0.96	0.77−1.18	0.674
**CD19^{+}CD27^{brt}CD38^{brt}**	1.01	0.97−1.06	0.622	1.03	0.94−1.14	0.493
**Belimumab use**	0.79	0.65−0.95	**0.014**	0.70	0.47−1.03	0.069
**Interaction**	0.97	0.88−1.06	0.487	1.05	0.86−1.28	0.625
**Baseline through week 24**						
**CD19^{+}CD20^{+}**	1.05	0.79−1.38	0.741	1.40	0.85−2.30	0.190
**Belimumab use**	0.83	0.68−1.01	0.058	0.69	0.46−1.02	0.064
**Interaction**	0.99	0.72−1.36	0.952	0.70	0.38−1.30	0.261
**CD19^{+}CD20^{+}CD27^{+}**	1.14	0.95−1.38	0.168	1.39	1.05−1.84	**0.022**
**Belimumab use**	0.83	0.67−1.03	0.092	0.73	0.47−1.15	0.173
**Interaction**	0.89	0.73−1.09	0.254	0.72	0.52−0.99	**0.044**
**CD19^{+}CD20^{+}CD69^{+}**	0.99	0.93−1.05	0.692	1.01	0.90−1.12	0.899
**Belimumab use**	0.80	0.66−0.97	**0.026**	0.67	0.45−1.00	**0.047**
**Interaction**	1.02	0.95−1.10	0.525	1.01	0.88−1.17	0.876
**CD19^{+}CD20^{+}CD27^{-}**	1.01	0.75−1.37	0.924	1.27	0.71−2.26	0.416
**Belimumab use**	0.83	0.67−1.02	0.077	0.65	0.42−1.02	0.059
**Interaction**	0.99	0.69−1.44	0.970	0.71	0.33−1.53	0.384
**CD19^{+}CD20^{+}CD138^{+}**	0.98	0.92−1.04	0.478	1.02	0.90−1.14	0.801
**Belimumab use**	0.81	0.66−0.98	**0.035**	0.67	0.45−0.99	**0.045**
**Interaction**	1.04	0.96−1.13	0.357	0.94	0.79−1.13	0.520
**CD19^{+}CD20^{-}CD138^{+}**	0.98	0.90−1.08	0.725	1.05	0.90−1.22	0.558
**Belimumab use**	0.82	0.67−1.00	**0.047**	0.69	0.46−1.02	0.059
**Interaction**	1.01	0.91−1.13	0.828	0.92	0.74−1.15	0.469
**CD19^{+}CD20^{-}CD27^{brt}**	0.99	0.90−1.09	0.883	1.15	1.00−1.33	0.051
**Belimumab use**	0.81	0.66−0.99	**0.038**	0.70	0.46−1.05	0.085
**Interaction**	1.02	0.91−1.14	0.725	0.93	0.77−1.13	0.461
**CD19^{+}CD27^{brt}CD38^{brt}**	0.97	0.89−1.05	0.434	1.03	0.89−1.20	0.661
**Belimumab use**	0.80	0.65−0.98	**0.033**	0.63	0.42−0.96	**0.033**
**Interaction**	1.07	0.97−1.18	0.175	1.12	0.93−1.34	0.223
**Baseline through week 24 adj. for baseline through week 8**						
**CD19^{+}CD20^{+}**	1.09	0.81−1.48	0.553	1.81	1.08−3.05	**0.024**
**Belimumab use**	0.79	0.65−0.97	**0.024**	0.73	0.48−1.10	0.130
**Interaction**	0.98	0.70−1.36	0.883	0.70	0.38−1.30	0.255
**CD19^{+}CD20^{+}CD27^{+}**	1.17	0.96−1.42	0.125	1.58	1.18−2.11	**0.002**
**Belimumab use**	0.80	0.64−1.01	0.055	0.83	0.52−1.33	0.440
**Interaction**	0.89	0.73−1.10	0.280	0.73	0.53−1.01	0.054
**CD19^{+}CD20^{+}CD69^{+}**	0.99	0.93−1.05	0.748	1.02	0.91−1.15	0.703
**Belimumab use**	0.76	0.62−0.93	**0.008**	0.64	0.42−0.96	**0.030**
**Interaction**	1.01	0.94−1.09	0.726	1.01	0.88−1.17	0.872
**CD19^{+}CD20^{+}CD27^{-}**	1.06	0.76−1.48	0.725	1.74	0.93−3.28	0.084
**Belimumab use**	0.79	0.64−0.98	**0.036**	0.69	0.44−1.09	0.109
**Interaction**	0.97	0.66−1.43	0.887	0.71	0.32−1.55	0.387
**CD19^{+}CD20^{+}CD138^{+}**	0.96	0.90−1.03	0.284	1.04	0.91−1.19	0.583
**Belimumab use**	0.77	0.63−0.94	**0.009**	0.67	0.45−1.01	0.055
**Interaction**	1.03	0.95−1.12	0.446	0.94	0.78−1.13	0.528
**CD19^{+}CD20^{-}CD138^{+}**	0.95	0.87−1.05	0.315	0.98	0.83−1.16	0.788

**Belimumab use**	0.79	0.64−0.96	**0.019**	0.71	0.47−1.07	0.099
**Interaction**	1.02	0.91−1.14	0.784	0.90	0.71−1.13	0.349
**$CD19^+CD20^-CD27^{brt}$**	0.97	0.88−1.07	0.553	1.15	0.99−1.33	0.068
**Belimumab use**	0.77	0.63−0.94	**0.011**	0.71	0.46−1.08	0.106
**Interaction**	1.01	0.90−1.13	0.887	0.88	0.72−1.08	0.228
**$CD19^+CD27^{brt}CD38^{brt}$**	0.96	0.87−1.05	0.328	1.06	0.90−1.25	0.473
**Belimumab use**	0.76	0.62−0.94	**0.010**	0.63	0.41−0.97	**0.037**
**Interaction**	1.07	0.96−1.18	0.219	1.10	0.92−1.32	0.310

Data are presented as the hazard ratio (HR), 95% confidence interval (CI) and P value. Percentage changes above the 97.5^{th} percentile of the respective distribution were treated as outliers and were set to the value corresponding to the 97.5^{th} percentile. Adjusted models included relative to baseline percentage changes for each one of the investigated B cell subsets, age, sex, ancestry, SLE disease duration, belimumab use, the interaction between belimumab use and cell changes, azathioprine use, mycophenolate mofetil or sodium use, methotrexate use, other immunosuppressant use (cyclosporine, oral cyclophosphamide, leflunomide, mizoribine or thalidomide) and BLISS study. One set of models with any flare from week 24 through week 76 as the outcome additionally included B cell subset percentage changes from baseline through week 8 among covariates. Significant P values are in bold.
